# Mechanosensitive channels TMEM63A and TMEM63B mediate lung inflation–induced surfactant secretion

**DOI:** 10.1172/JCI174508

**Published:** 2023-12-21

**Authors:** Gui-Lan Chen, Jing-Yi Li, Xin Chen, Jia-Wei Liu, Qian Zhang, Jie-Yu Liu, Jing Wen, Na Wang, Ming Lei, Jun-Peng Wei, Li Yi, Jia-Jia Li, Yu-Peng Ling, He-Qiang Yi, Zhenying Hu, Jingjing Duan, Jin Zhang, Bo Zeng

**Affiliations:** 1 Key Laboratory of Medical Electrophysiology, Ministry of Education and Medical Electrophysiological Key Laboratory of Sichuan Province, Institute of Cardiovascular Research, and; 2 Department of Cardiothoracic Surgery, The Affiliated Traditional Chinese Medicine Hospital, Southwest Medical University, Luzhou, Sichuan, China.; 3 Human Aging Research Institute and School of Life Sciences and; 4 School of Basic Medical Sciences, Nanchang University, Nanchang, Jiangxi, China.

**Keywords:** Pulmonology, Ion channels, Pulmonary surfactants

## Abstract

Pulmonary surfactant is a lipoprotein complex lining the alveolar surface to decrease the surface tension and facilitate inspiration. Surfactant deficiency is often seen in premature infants and in children and adults with respiratory distress syndrome. Mechanical stretch of alveolar type 2 epithelial (AT2) cells during lung expansion is the primary physiological factor that stimulates surfactant secretion; however, it is unclear whether there is a mechanosensor dedicated to this process. Here, we show that loss of the mechanosensitive channels TMEM63A and TMEM63B (TMEM63A/B) resulted in atelectasis and respiratory failure in mice due to a deficit of surfactant secretion. TMEM63A/B were predominantly localized at the limiting membrane of the lamellar body (LB), a lysosome-related organelle that stores pulmonary surfactant and ATP in AT2 cells. Activation of TMEM63A/B channels during cell stretch facilitated the release of surfactant and ATP from LBs fused with the plasma membrane. The released ATP evoked Ca^2+^ signaling in AT2 cells and potentiated exocytic fusion of more LBs. Our study uncovered a vital physiological function of TMEM63 mechanosensitive channels in preparing the lungs for the first breath at birth and maintaining respiration throughout life.

## Abstract 2

In alveolar epithelial cells TMEM63A/B channels act as mechanosensors to mediate lung expansion-induced surfactant release, which is required for breath from birth.

## Graphical abstract

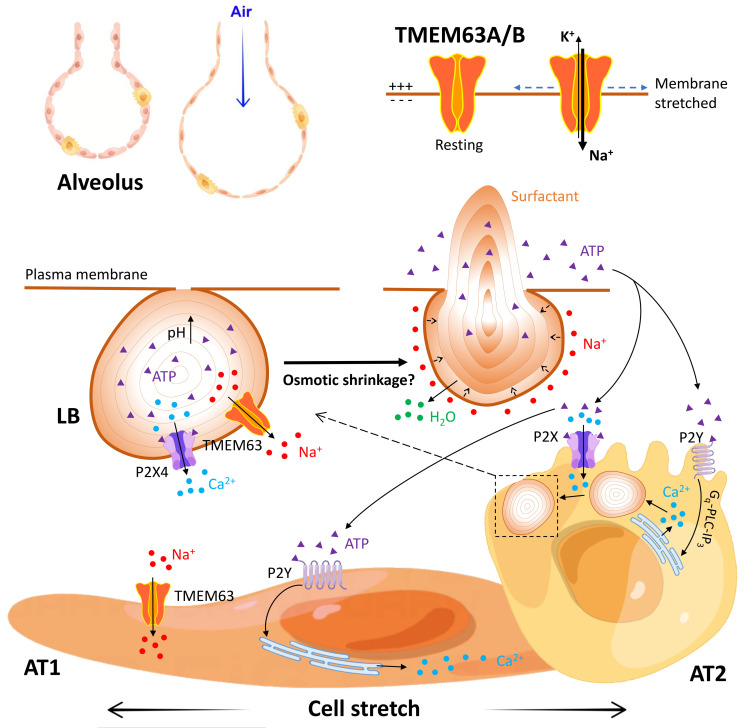

## Introduction

Pulmonary surfactant is necessary for lowering surface tension of alveolar lining liquid and effective inspiration (1). Deficiency of pulmonary surfactant results in neonatal respiratory distress syndrome in premature infants (1) and is involved in a variety of pulmonary diseases in adults including acute respiratory distress syndrome, obstructive lung diseases, respiratory infections, and pulmonary fibrosis (2). Inflation of the lung is known to stimulate pulmonary surfactant secretion via mechanosensitive ATP release and Ca^2+^-dependent pathways in alveolar epithelial cells (AECs) (3, 4). AECs consist of large and thin alveolar type 1 (AT1) cells for gas exchange and cuboidal alveolar type 2 (AT2) cells for synthesis and secretion of pulmonary surfactant. In AT2 cells, pulmonary surfactant and ATP are stored in lamellar bodies (LBs), a specialized lysosome-related organelle (5).

A commonly accepted mechanism of lung inflation–induced surfactant secretion is that AEC stretching causes ATP release into the alveolar surface, and then this released ATP evokes Ca^2+^ signaling in AT2 cells, which promotes the fusion of LBs to the plasma membrane and surfactant exocytosis (3, 4). However, several critical questions have yet to be addressed. (a) How does mechanical stretch induce ATP release? Both AT1 and AT2 cells have been proposed to be the sources of ATP by in vitro studies (6, 7), and the pathway of ATP release has not been consistently illustrated. (b) Why is ATP treatment a potent stimulator of LB fusion to the plasma membrane, but a much less efficient facilitator of surfactant release than cell stretch (8)? (c) Are the stretch-induced Ca^2+^ signals only a response of P2 purinergic receptors to extracellular ATP, or do they contain a component contributed by a Ca^2+^-permeable mechanosensitive channel directly activated by cell stretch?

To address these questions, it is most important to understand how mechanical stretch is sensed and transduced in AECs. It remains to be determined whether there is a primary mechanosensor in AECs that initiates or controls the release of ATP and surfactant. Among various cellular structures and proteins that are capable of sensing mechanical forces, mechanosensitive ion channels are most relevant to Ca^2+^ signaling and exocytosis. Currently, only 4 types of mammalian ion channels are generally accepted as bona fide mechanically activated channels, including Piezo1/2, two-pore domain K^+^ channels (K_2P_, also known asTREK/TRAAK), transmembrane channel–like TMC1/2, and transmembrane 63 (TMEM63) channels (9–11). Although Piezo1 has been found to regulate ATP release in an AT1 cell line (12), its functional expression in AECs is not detected in mouse lungs in vivo (13). K_2P_ channels are widely expressed but not directly related to Ca^2+^ signaling due to their repolarizing action (14). TMC1 and TMC2 form mechanotransduction channels in cochlear hair cells, and their mutations cause only hearing loss (15). As a most recently identified mechanosensitive channel (11, 16, 17), TMEM63B plays a critical role in hair cell survival and is essential for hearing (18), but the physiological functions of TMEM63A, TMEM63B, and TMEM63C (TMEM63A/B/C) in other systems are unclear.

To elucidate the mechanosensitive pathways underlying alveolar distention–induced surfactant secretion, we investigated mechanical ventilation–induced Ca^2+^ transients in intact lungs and stretch-activated membrane conductance in ACEs. We demonstrate that the mechanosensitive channels TMEM63A/B are mechanosensors in AECs that mediate lung inflation–induced release of surfactant and ATP and show their essential roles in maintaining respiratory function.

## Results

### Lung inflation–induced, ATP-mediated Ca^2+^ transients in alveolar cells.

**Figure 1:**
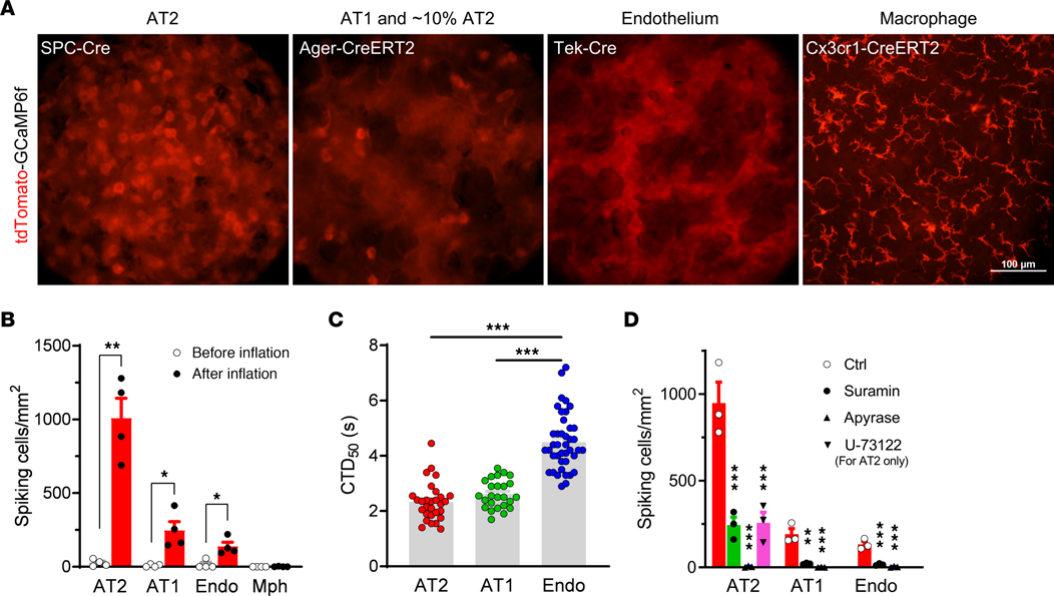
Mechanical ventilation–induced Ca^2+^ transients in alveolar cells from ex vivo mouse lungs. (**A**) tdTomato fluorescence in ex vivo mouse lungs with Cre-driven expression of tdTomato-GCaMP6f in different cell types. Scale bars: 100 μm. (**B**) Density of cells with Ca^2+^ transients (spiking cells, indicated by sharp changes in GCaMP6f intensity) before and after mechanical ventilation with a tidal volume of 200 μL for 5 minutes. *n* = 4 lung lobes. (**C**) Duration (seconds) of Ca^2+^ transients above 50% peak value (CTD_50_). *n* = 29, 25, and 41 for AT2, AT1, and endothelial cells, respectively. (**D**) Density of spiking cells in lungs intratracheally instilled with 100 μL vehicle (0.2% DMSO), suramin (300 μM; blocking P2X and P2Y receptors), apyrase (10 U/mL; hydrolyzing ATP), or U-73122 (20 μM; inhibiting P2Y-G_q_-PLC-IP_3_R pathway) and ventilated with a tidal volume of 200 μL for 5 minutes. *n* = 3 lung lobes. *P < 0.05, **P < 0.01, and ***P < 0.001, by 2-tailed, unpaired Student’s *t* test (**B**), or 1-way ANOVA with Tukey’s test (**C** and **D**). Endo, endothelial cells; Mph, macrophages.

Using a Cre-dependent tdTomato-GCaMP6f–expressing mouse line (19), we examined Ca^2+^ signals in different alveolar cell types after mechanical ventilation in intact lungs ex vivo. GCaMP6f is a GFP-based Ca^2+^ indicator and tdTomato is a Ca^2+^-insensitive red fluorescent protein fused to GCaMP6f as a reference for expression and localization. In the lung, SPC-Cre, Tek-Cre, and Cx3cr1-CreERT2 facilitate relatively specific expression of tdTomato-GCaMP6f in AT2 cells, endothelial cells, and myeloid (mostly macrophages) cells, respectively, while Ager-CreERT2 is functional in most AT1 cells and approximately 10% of AT2 cells (Figure 1A and Supplemental Figure 1; supplemental material available online with this article; https://doi.org/10.1172/JCI174508DS1). Post-inflation Ca^2+^ transients were observed in AT2, AT1, and endothelial cells, but not in macrophages (Figure 1B and Supplemental Videos 1–5). The Ca^2+^ transients were most evident in AT2 cells and relatively sparse in AT1 and endothelial cells (Figure 1B); however, the duration of each Ca^2+^ transient was longer in endothelial cells than in AT1 and AT2 cells (Figure 1C). The Ca^2+^ transients were significantly suppressed by suramin, a nonselective inhibitor of P2 purinergic receptors, and abolished by apyrase, an ATP diphosphohydrolase (Figure 1D), suggesting that they were triggered by ATP that was released to the alveolar surface upon mechanical ventilation. Additionally, the phospholipase C (PLC) inhibitor U-73122 also robustly reduced Ca^2+^ transients in AT2 cells (Figure 1D), confirming the involvement of P2Y receptors via the G_q_-PLC-IP_3_ receptor (IP_3_R) pathway (20, 21). The consistent action of suramin, apyrase, and U-73122 largely excluded the ATP-independent off-target effects of suramin and U-73122 (22, 23). However, in AT2 cells, genetic ablation of P2 receptors such as P2X4, P2X7, P2Y2, and P2Y6 (24) would provide more substantial evidence for their contribution to lung inflation–induced Ca^2+^ signals.

**Figure 2:**
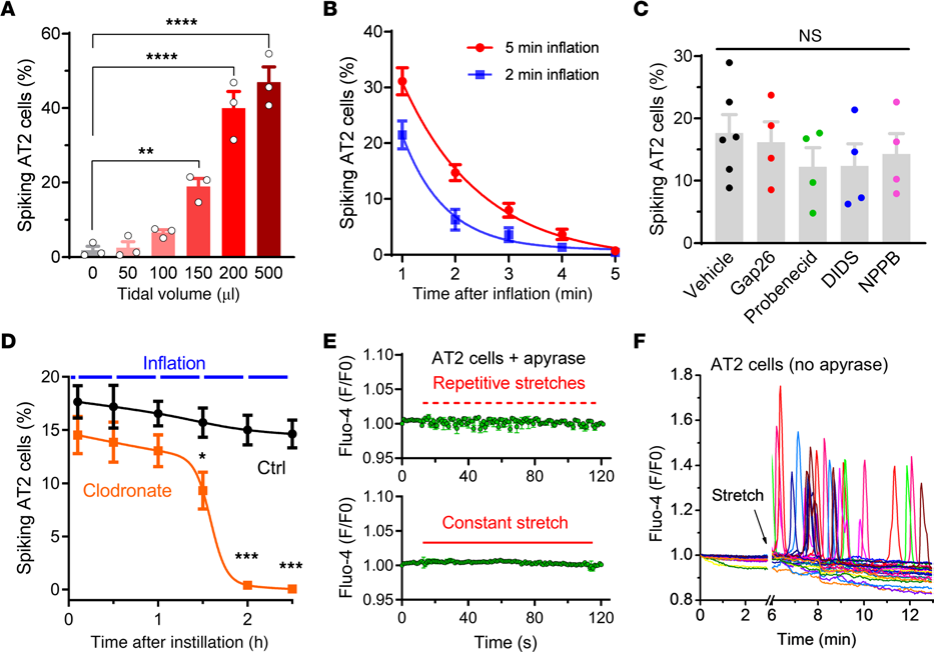
Properties of lung inflation– and stretch-induced Ca^2+^ transients in AT2 cells. (**A**) Density of spiking AT2 cells in lungs ventilated with different tidal volumes for 5 minutes. The number of GCaMP6f-spiking cells was divided by the number of tdTomato^+^ cells (i.e., total number of AT2 cells) in the same area to calculate the percentage. *n* = 3 lung lobes. **P < 0.01 and ****P < 0.0001, by 1-way ANOVA with Tukey’s test. (**B**) Rapid decay of spiking AT2 cell density after ventilation with a tidal volume of 200 μL for 2 minutes and 5 minutes, respectively. Data were fitted with a single exponential decay function. *n* = 5 lung lobes. (**C**) Percentage of spiking AT2 cells in lungs instilled with 150 μL vehicle (0.1% DMSO), the Cx43 blocker Gap26 (500 μM), the pannexin blocker probenecid (2 mM), the Cl^–^ channel blocker DIDS (200 μM), or NPPB (100 μM) after ventilation with a tidal volume of 150 μL for 5 minutes. *n* = 6 (vehicle) and 4 (others) lung lobes. NS, by 1-way ANOVA. (**D**) Percentage of spiking AT2 cells in lungs instilled with 150 μL clodronate (100 μM) or control solution. The data were fitted with a biphasic dose-response curve (corresponding to the abundance of ATP-containing LBs). *n* = 6 lung lobes. *P < 0.05 and ***P < 0.001, by 2-way ANOVA with Šidák’s test. (**E**) Lack of a direct stretch-activated Ca^2+^ response in primary AT2 cells cultured on an elastic membrane. Apyrase (10 U/mL) was used to eliminate ATP released into the extracellular space. *n* = 12 cells. (**F**) Stretch-induced, ATP-mediated Ca^2+^ oscillations in primary AT2 cells. Each trace represents fluorescence of an AT2 cell. *n* = 26 cells.

The density of AT2 cells showing Ca^2+^ transients (“spiking AT2”) increased with larger tidal volumes within the physiological range (Figure 2A). Inflation for 5 minutes resulted in more Ca^2+^ transients in AT2 cells than a 2-minute inflation (Supplemental Figure 2A), and the percentage of spiking AT2 cells experienced exponential decay and diminished to basal levels within 5 minutes after inflation (Figure 2B).

We then explored possible pathways of ATP release in the lungs. Gap junctional hemichannels, volume-regulated anion channels (VRACs), and Maxi Cl^–^ channels are proposed ATP-conducting channels widely expressed in many organs, including the lung (3). Blockers of gap junctions (Gap26 for Cx43, the dominant connexin isoform in the lung, and probenecid for pannexins) and Cl^–^ channels (DIDS and NPPB, nonselective for VRAC and Maxi Cl^–^) did not significantly affect inflation-induced Ca^2+^ transients in AT2 cells (Figure 2C). The nonselective gap junctional blocker carbenoxolone (CBX) completely abolished the Ca^2+^ transients (Supplemental Figure 2B), however, this turned out to be the consequence of its off-target inhibition of purinergic receptors, as evidenced by direct instillation of ATP into the lung (Supplemental Figure 2C) and the Ca^2+^ response of A549 lung cancer cells to different concentrations of extracellular ATP (Supplemental Figure 2D).

To test LB exocytic ATP release, we used clodronate, an inhibitor of vesicular nucleotide transporter (VNUT) (25), to block ATP transport into LBs (5). Ex vivo lungs were mechanically ventilated for 2.5 hours immediately after instillation of clodronate and stopped for imaging of Ca^2+^ in AT2 cells every 30 minutes. The density of spiking AT2 cells with clodronate showed a slow decrease similar to that seen in the control group within 1 hour, which was followed by a dramatic decline that almost eliminated the Ca^2+^ spikes after 2 hours (Figure 2D). This biphasic decline could be attributed to depletion of ATP-containing LBs by inflation-induced exocytosis after treatment with clodronate for 1 hour. The remaining LBs may still have been able to release surfactant, however, the deficiency of exocytic ATP would no longer stimulate Ca^2+^ response in the cells. These results suggest that exocytosis of LBs is a probable pathway of mechanosensitive ATP release in AT2 cells.

Last, we used ultrafast imaging to detect Ca^2+^ signals in AT2 cells directly triggered by membrane stretch. However, we did not observe a notable occurrence of Ca^2+^ transients immediately with the first pulse of mechanical ventilation (Supplemental Video 6). When the lung was preinstilled with apyrase, only rarely occurring Ca^2+^ transients (presumably due to incomplete hydrolysis of extracellular ATP) were observed during a 5-minute constant inflation with a tidal volume of 500 μL (Supplemental Video 7). In line with this, either repetitive or constant stretch of the elastic substrate of cultured primary AT2 cells failed to induce a direct Ca^2+^ response in the presence of apyrase (Figure 2E and Supplemental Videos 8 and 9). In contrast, Ca^2+^ oscillations could be readily elicited by stretch in the absence of apyrase (Figure 2F and Supplemental Video 10). These results suggested a lack of highly Ca^2+^-permeable mechanosensitive channels in AT2 cells.

### Mechanosensitive currents in AT1 and AT2 cells.

To investigate the mechanosensitive electrical activity of AECs, we first performed blunt-probe indentation on primarily cultured AT2 cells. Since the cells were loosely adhered to fibronectin-coated coverslips, most of them were pushed away once in contact with the probe and did not show convincing mechanosensitive currents before losing the gigaseal. Considering that cell indentation is not a physiological way of mechanical stimulation for cells in the lung, while membrane stretch is more relevant, we turned to using pressure clamp to examine the mechanosensitivity of AECs.

**Figure 3:**
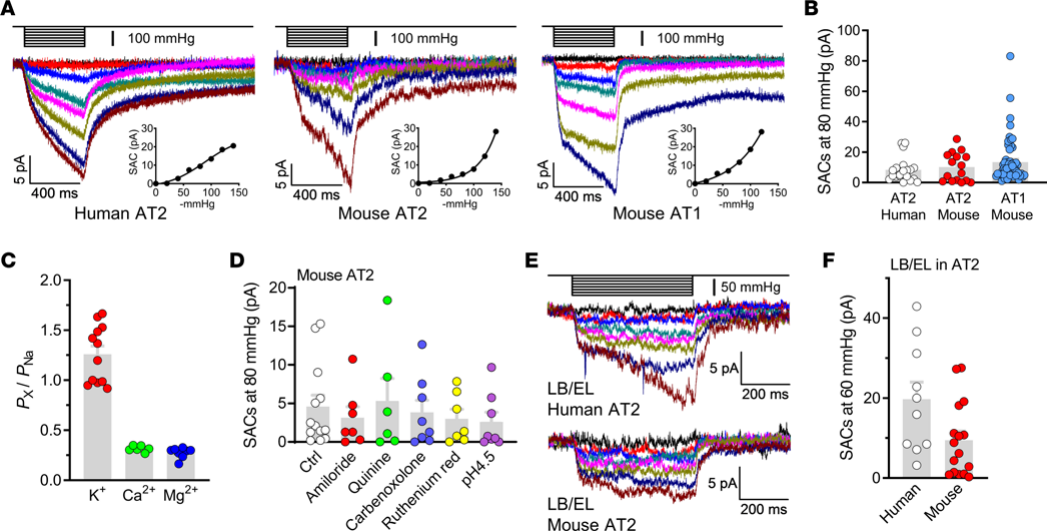
Mechanosensitive currents in human and mouse AECs. (**A**) Stretch-activated currents (SACs) under a cell-attached configuration in primary human and mouse AT2 cells and mouse AT1 cells in acute lung slices. The holding potential was –80 mV, and the vacuum pressures were applied to the clamped membrane with a –20 mmHg increase for each step. Insets are pressure-current relationships of the corresponding recordings. (**B**) Amplitudes of SACs induced by –80 mmHg pressure in human or mouse AT2 cells and mouse AT1 cells. *n* = 20, 16, and 56 for human AT2 cells, mouse AT2 cells, and mouse AT1 cells, respectively. (**C**) Relative permeability of K^+^, Ca^2+^, and Mg^2+^ versus Na^+^ for stretch-activated currents in mouse AT2 cells. *n* = 12, 6, and 8 cells for K^+^, Ca^2+^, and Mg^2+^, respectively. (**D**) Nonselective blockers of ENaC (amiloride, 10 μM), K^+^ channels (quinine, 500 μM), gap junctions (CBX, 100 μM), and Piezo1 (ruthenium red, 50 μM), and acidic pH did not affect the stretch-activated currents in mouse AT2 cells. *n* = 13, 7, 6, 8, 7, and 8 cells from left to right. (**E**) SACs under vesicle-attached configuration in enlarged LBs and ELs (LB/EL) from mouse AT2 cells. The holding potential was –60 mV, and the vacuum pressures were applied with a –10 mmHg increase for each step. (**F**) Comparison of the amplitudes of stretch-activated currents from LB/EL. *n* = 10 and 16 human and mouse cells, respectively.

In pressure clamp in the cell-attached mode, we observed membrane stretch–induced macroscopic currents for AT2 cells in culture and AT1 cells in acute lung slices, with similar properties, including slow activation, no inactivation, and pressure dependence (Figure 3A). Because in most recordings the currents increased exponentially within the pressure range of 0 to –140 mmHg and did not reach a plateau phase (Figure 3A, insets), the half-activation pressures (*P*_50_) were not calculated. The amplitudes of currents were comparable between human and mouse AT2 cells, whereas a small subset of mouse AT1 cell recordings exhibited much larger currents (Figure 3B). For both human and mouse AT2 cells, cell-attached recordings with different cations in the pipettes showed that the mechanosensitive currents mainly consisted of K^+^ and Na^+^ conductance, whereas Ca^2+^, Mg^2+^ or NMDG^+^ almost eliminated the inward currents (Supplemental Figure 3, A and B). The permeability of K^+^ was slightly higher than that of Na^+^, and the permeability of Ca^2+^ and Mg^2+^ was much lower (Figure 3C). We confirmed the dramatically higher permeability of Na^+^ and K^+^ compared with Ca^2+^ by inside-out recordings (Supplemental Figure 3C). At negative membrane potentials of –27 to –63 mV in primary AT2 cells (26), the stretch-activated ion flow should be mainly Na^+^ influx rather than K^+^ efflux.

We also detected single-channel activities in some recordings on AT2 cells, which are presumably epithelial Na^+^ channel (ENaC) currents that have been widely recognized (27). However, these single-channel currents are insensitive to membrane stretch (Supplemental Figure 3D), and the above-mentioned stretch-activated macroscopic currents were not observed in these recordings, suggesting that these 2 types of channels do not coexist in the same membrane patches. To investigate the identity of the ion channel that generates the stretch-activated currents, we used nonselective blockers for ENaC (amiloride), 2-pore domain K^+^ channels (quinine), gap junctional hemichannels (CBX), and Piezo channels (ruthenium red) in the pipette and bath solutions, however, none of these agents had a significant effect (Figure 3D), indicating that the stretch-activated currents we observed were not generated by channels sensitive to these blockers, including the 4 mentioned above. The currents were also insensitive to acidic extracellular pH (Figure 3D), suggesting that they were not conducted by proton channels, acid-sensitive cation channels (ASICs), or proton-activated Cl^–^ channels (PACs).

Given the unusual large size of LBs (0.1–2.4 μm) (28), they are very likely to be mechanically stressed during the stretch of AT2 cells. Therefore, we also examined the mechanosensitivity of the endolysosomal system in AT2 cells, the overwhelming majority of which are LBs. The small molecule vacuolin-1 was used to induce fusion of LB/EL into giant vesicles large enough (>4 μm) for patch clamping (29). The enlarged LB/EL vesicles in AT2 cells were isolated and pressure clamped under vesicle-attached configuration. The mechanosensitive currents of LB/EL vesicles were evident, with slow-activating and noninactivating properties similar to those of the plasma membrane currents (Figure 3E). However, the current amplitudes of LB/EL vesicles measured at –60 mV/–60 mmHg were comparable to the plasma membrane currents at –80 mV/–80 mmHg (Figure 3, B and F), suggesting that LBs were more electrically sensitive to membrane stretch.

### TMEM63A/B KO results in atelectasis, pulmonary edema, and respiratory failure.

In search of the molecular identity of stretch-activated ion channels in AECs, we found that TMEM63B, a recently identified mechanosensitive channel (16, 17), was highly expressed in both AT1 and AT2 cells, according to single-cell RNA-Seq data from human and mouse lungs (Supplemental Figure 4). TMEM63A, a close homolog of TMEM63B, was also expressed in AT1 and AT2 cells at relatively lower levels (Supplemental Figure 4).

**Figure 4:**
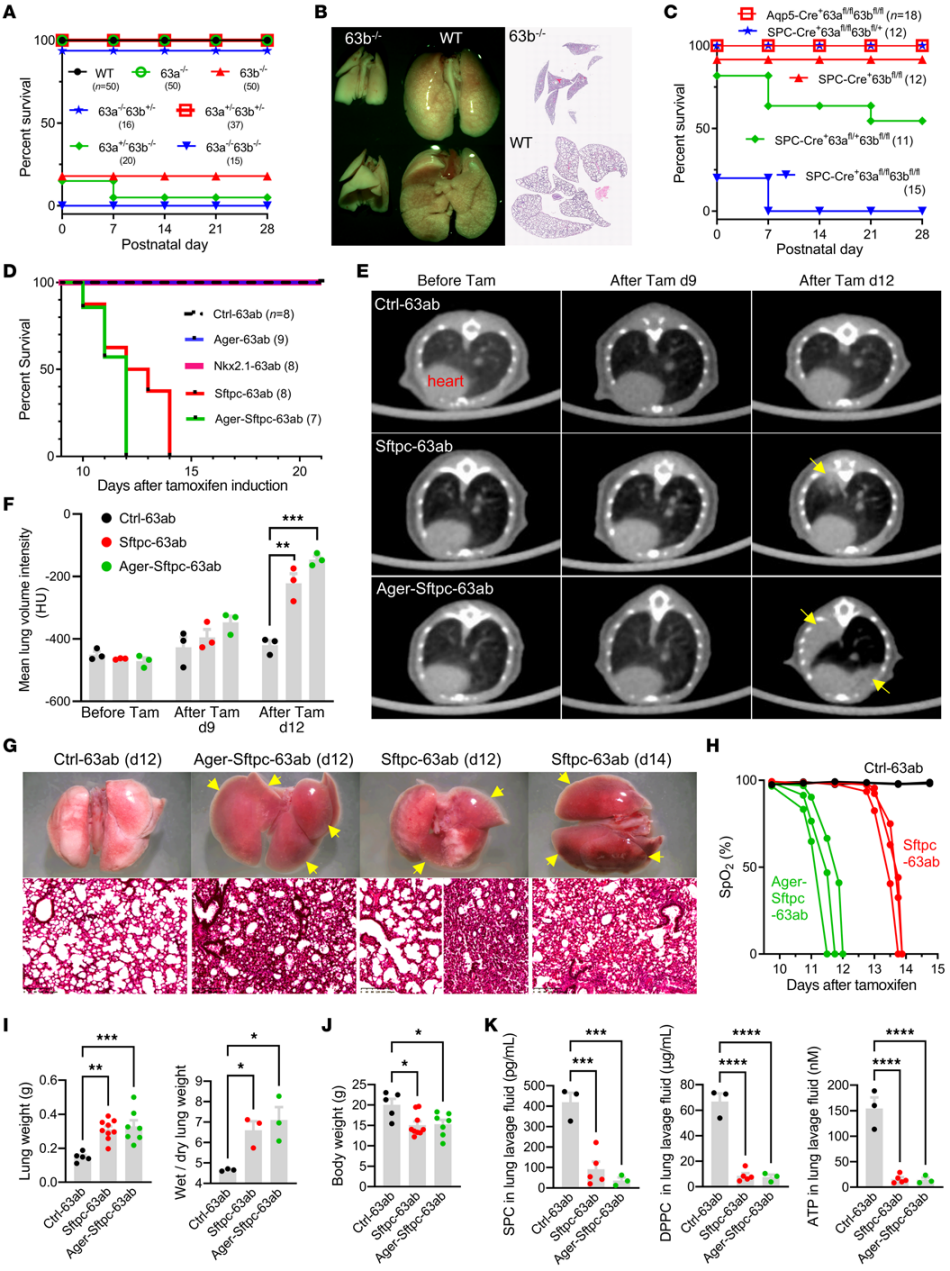
Lethal pulmonary phenotypes of *Tmem63a/b*-KO mice. (**A**) Survival curves for constitutive *Tmem63a/b*-KO mice of different genotypes. The numbers of mice are shown in parentheses. (**B**) Failure of alveolar expansion in *Tmem63b*-KO mice after birth (P0). Original magnification, ×2. (**C**) Survival curves of AEC-specific *Tmem63a/b* conditional-KO mice with different genotypes. Aqp5-Cre was expressed in AT1 cells and approximately 50% of AT2 cells SPC-Cre was expressed in all AT2 cells. (**D**) Survival curves for tamoxifen-inducible, AEC-specific *Tmem63a/b*-cDKO mice. Ager-CreERT2 was expressed in AT1 cells and approximately 10% of AT2 cells; Nkx2.1-CreERT2 was expressed in AT1 cells and approximately 80% of AT2 cells; and Sftpc-CreERT2 was expressed in all AT2 cells. Ctrl-63ab represents *63a^fl/fl^* *63b^fl/fl^* mice without Cre, and others are with the corresponding CreERT2. (**E**) Micro-CT images of mouse lungs after tamoxifen induction. Arrows indicate regions of atelectasis. (**F**) Mean lung volume intensities for mice before and after tamoxifen induction, measured from the micro-CT images. *n* = 3 mice. (**G**) Atelectasis in cDKO mice illustrated by freshly dissected lungs and H&E-stained sections. Arrows indicate collapsed lobes. Scale bars: 200 μm. (**H**) A dramatic decline of SpO_2_ before respiratory failure was observed in cDKO mice. *n* = 3 mice. (**I**) Characteristics of pulmonary edema in cDKO mice at post-tamoxifen day 12. *n* = 5, 9, and 7 mice for lung weight from left to right; *n* = 3 mice for wet/dry ratio. (**J**) Body weights in cDKO mice at post-tamoxifen day 12. *n* = 5, 9, and 7 mice from left to right. (**K**) Deficiency of secreted SPC, surfactant phospholipid DPPC, and ATP in BALF collected from cDKO mice at post-tamoxifen day 10. *n* = 3, 5, and 3 mice from left to right in each graph. *P < 0.05, **P < 0.01, ***P < 0.001, and ****P < 0.0001, by 1-way ANOVA with Tukey’s test.

To explore the physiological function of TMEM63B, we generated constitutive *Tmem63b*-KO (*63b^–/–^*) mice and found that most (~82%) of the *63b^–/–^* mice died at birth without alveolar expansion (Figure 4, A and B, Supplemental Table 1, and Supplemental Figure 5). However, like their WT and *63b^+/–^* littermates, the remaining *63b^–/–^* neonates that were able to breathe all survived (Figure 4A), with normal lung structure and function (Supplemental Figure 5). Considering the coexpression of TMEM63A/B in most tissues, we suspected that TMEM63A would complement the function of TMEM63B when it was absent. Constitutive *Tmem63a*-KO (*63a^–/–^*) mice were all viable from the neonatal to late adult stages (Figure 4A and Supplemental Table 1). Combined deletion of *Tmem63a* and *Tmem63b* resulted in the death of all *63a^–/–^* *63b^–/–^* neonates, with the same phenotype as the *63b^–/–^* mice that died at birth (Figure 4A, Supplemental Table 1, and Supplemental Figure 5).

To validate the roles of TMEM63A/B in AECs, we generated *Tmem63a* and *Tmem63b* conditional double-KO (*Tmem63a*/*b-*cDKO) mice with gene deletions in AT1 and AT2 cells using Aqp5-Cre and SPC-Cre, respectively. Deficiency of *Tmem63a*/*b* in AT1 cells and in approximately 50% of the AT2 cells (*Aqp5-Cre^+/–^* *63a^fl/fl^* *63b^fl/fl^*, referred to hereafter as Aqp5-63ab), Supplemental Figure 1) did not affect the viability of mice at any developmental stages (Figure 4C, Supplemental Table 1, and Supplemental Figure 5). In contrast, only approximately 20% of the *SPC-Cre^+/–^* *63a^fl/fl^* *63b^fl/fl^* mice (SPC-63ab, with Cre activity in all AT2 cells) survived at P0, but all died before P7 as a result of respiratory failure (Figure 4C, Supplemental Table 1, and Supplemental Figure 5). We also generated *Lyz2-Cre^+/–^* *63a^fl/fl^* *63b^fl/fl^* (Lyz2-63ab) mice using Lyz2-Cre that was expressed in macrophages and in approximately 6% of AT2 cells (Supplemental Figure 1); however, we observed no respiratory phenotype for these mice (Supplemental Table 1 and Supplemental Figure 5).

To investigate the function of TMEM63A/B in alveolar epithelium at the adult stage, we bred *Tmem63a*/*b-*cDKO mice with tamoxifen-inducible gene deletions in AT1 and AT2 cells using Ager-CreERT2 (*Ager-63ab*) and Sftpc-CreERT2 (Sftpc-63ab), respectively. To achieve *Tmem63a*/*b-*cDKO in both AT1 and AT2 cells, we generated double–*Cre* *Ager-CreERT2^+/–^* *Sftpc-CreERT2^+/–^* *63a^fl/fl^* *63b^fl/fl^* (Ager-Sftpc-63ab) mice and *Nkx2.1-CreERT2^+/–^* *63a^fl/fl^* *63b^fl/fl^* (Nkx2.1-63ab) mice with Cre activity in AT1 cells and approximately 80% of AT2 cells (Supplemental Figure 1). Ager-63ab mice did not show any abnormal phenotype after tamoxifen induction for 5 consecutive days (Figure 4D, Supplemental Table 1, and Supplemental Figure 5). Sftpc-63ab mice exhibited signs of atelectasis and progressed to respiratory failure from day 10 after the last tamoxifen injection, and all animals died no later than the 14th day (Figure 4, D–G, and Supplemental Table 1). Lung lobes in Sftpc-63ab mice collapsed gradually and could not be inflated by mechanical ventilation (Supplemental Video 11). Ager-Sftpc-63ab mice had an earlier onset of atelectasis and respiratory failure, with a maximal survival period of 12 days after tamoxifen administration (Figure 4, D–G, and Supplemental Table 1). However, Nkx2.1-63ab mice retained normal lung function and survived like their *63a^fl/fl^ 63b^fl/fl^* (Ctrl-63ab) littermates (Figure 4D, Supplemental Table 1, and Supplemental Figure 5). These results suggest that only complete ablation of *Tmem63a* and *Tmem63b* in all AT2 cells (Sftpc-63ab) could result in a lethal respiratory phenotype in mice. In Aqp5-63ab and Nkx2.1-63ab mice, the Cre^–^ AT2 cells with intact *Tmem63a* and *Tmem63b* could compensate for the loss of the 2 genes in Cre^+^ AT2 cells. Surfactant secreted from these Cre^–^ AT2 cells was sufficient to facilitate effective lung inflation during breathing.

All atelectasis-affected mice experienced a dramatic decline in saturation of peripheral oxygen (SpO_2_) that began 24 hours before death (Figure 4H). The lung weights were much higher in Sftpc-63ab and Ager-Sftpc-63ab mice than in the Ctrl-63ab littermates due to pulmonary edema (Figure 4I). Dysfunction of the lung ultimately resulted in a dramatic loss of body weight (Figure 4J). The concentrations of secreted SPC, 1,2-dipalmitoyl-sn-glycero-3-phosphocholine (DPPC, the major surfactant lipid), and ATP in bronchoalveolar lavage fluid (BALF) collected on day 10 after tamoxifen induction were significantly lower in Sftpc-63ab and Ager-Sftpc-63ab mice than in their Ctrl-63ab littermates (Figure 4K and Supplemental Figure 6). These in vivo data indicate that TMEM63A/B in AT2 cells were essential for surfactant secretion and that their activity in AT1 cells could further support normal lung function.

To examine whether *Tmem63a*/*b-*cDKO had caused the respiratory phenotype by compromising AT2 cell survival and LB biogenesis, we stained AT2 cells with 3 markers — LysoTracker, Pro-SPB, and Pro-SPC — in acute lung slices and paraffin sections, respectively. All 3 markers showed that the density of AT2 cells in the lungs was not affected by *Tmem63a*/*b-*cDKO (Supplemental Figure 7), although some differences were observed for the staining intensity of LysoTracker and Pro-SPC. We observed a larger number of AT2 cells with large and bright LysoTracker spots (LBs) in cDKO mice (Supplemental Figure 7A). The expression of Pro-SPB was comparable between control and cDKO mice (Supplemental Figure 7B). The staining intensity of Pro-SPC was significantly reduced in cDKO mice (Supplemental Figure 7C), which we found was actually caused by knockin of CreERT2 in the endogenous *Sftpc* locus, as the Sftpc-CreERT2 mice showed similar reductions of Pro-SPC (Supplemental Figure 7D). Because of the reduced expression of Pro-SPC, the level of secreted mature SPC was also lower in Sftpc-CreERT2 mice (this impairment was much weaker than that caused by *Tmem63a*/*b-*cDKO), however, the secretion of surfactant lipids was not affected (Supplemental Figure 7E). These results suggest that *Tmem63a*/*b-*cDKO did not change the identity or viability of AT2 cells, but rather caused enlargement and malfunction of LBs.

### TMEM63A/B are required for lung inflation–induced Ca^2+^ transients and surfactant release.

**Figure 5:**
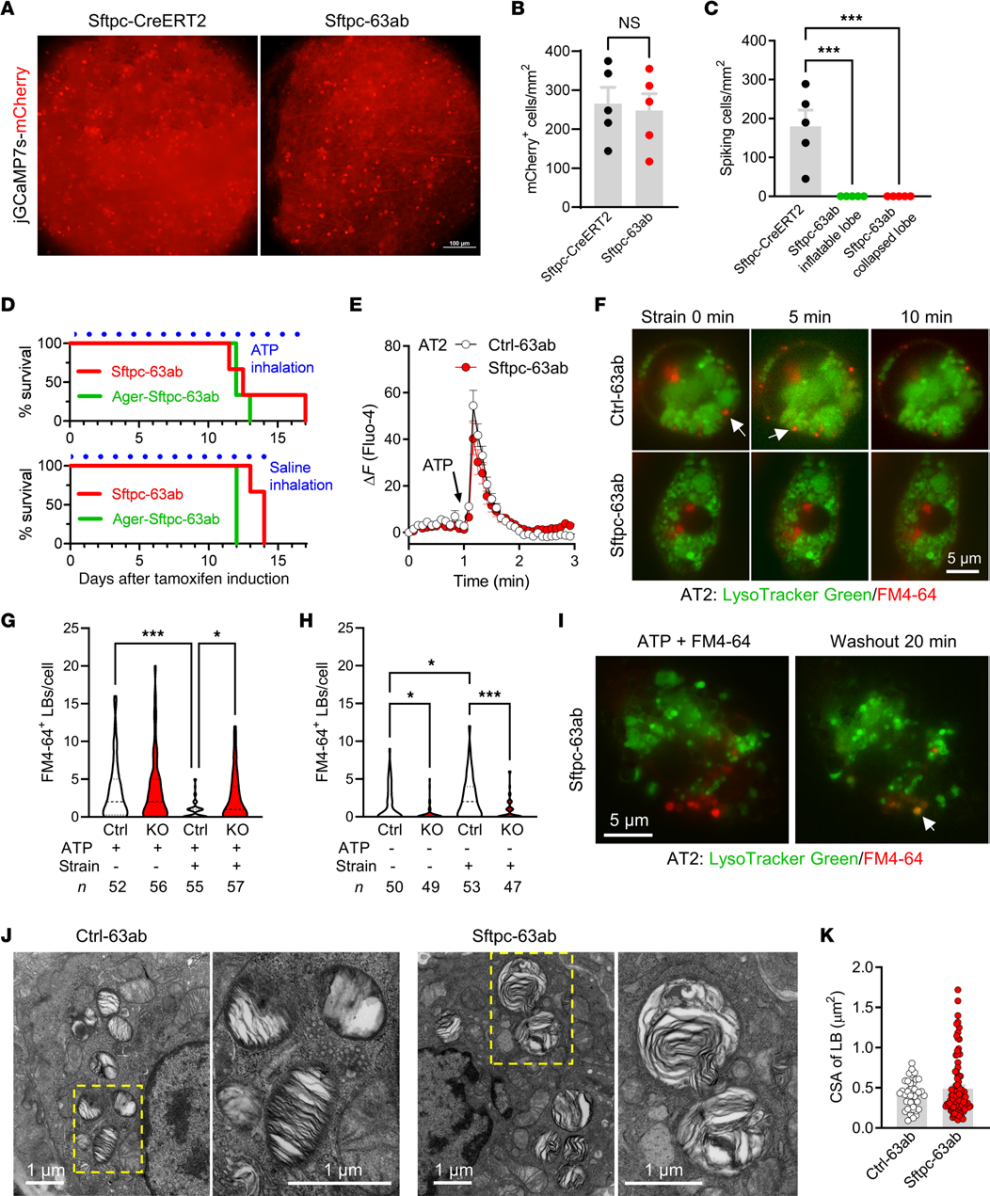
Deficiency of TMEM63A/B abolishes ventilation-induced Ca^2+^ transients and surfactant release in AT2 cells. (**A**) mCherry fluorescence in AAV-infected (CAG-DIO-jGCaMP7s-mCherry–infected) lungs showing positively transduced AT2 cells (brighter spots). Sftpc-63ab represents *Sftpc-CreERT2^+/–^* *63a^fl/fl^* *63b*^fl/fl^. Scale bar: 100 μm. (**B**) The densities of positively transduced AT2 cells were comparable between the control Sftpc-CreERT2 and Sftpc-63ab mice. *n* = 5 lung lobes. NS, nonsignificant, by 2-tailed, unpaired Student’s *t* test. (**C**) Lung inflation–induced Ca^2+^ transients in AT2 cells were completely abolished in all lung lobes from Sftpc-63ab mice, as revealed by jGCaMP7s fluorescence. *n* = 5 lung lobes. ***P < 0.001, by 1-way ANOVA with Tukey’s test. (**D**) Survival curves for *Tmem63a*/*b-*cDKO mice that received daily inhalation (30 min each time, 3 times/day) of aerosolized ATP (200 mM) or saline solution after tamoxifen induction. *n* = 3 mice. (**E**) ATP-induced Ca^2+^ response in primary AT2 cells isolated from Ctrl-63ab (*63a^fl/fl^ 63b^fl/fl^*) and Sftpc-63ab mice (*n* = 30 and 35 cells, respectively). (**F**) Cell strain–induced surfactant release occurred in AT2 cells from Ctrl-63ab, but not Sftpc-63ab, mice. Unfused LBs were stained by LysoTracker Green; LBs fused on the plasma membrane were positive for FM4-64. Arrows indicate LBs that released surfactant (FM4-64 fluorescence disappeared after strain). Scale bar: 5 μm. (**G** and **H**) *Tmem63a*/*b-*cDKO did not affect ATP-induced LB fusion but significantly attenuated cell strain–induced surfactant release. Ctrl, Ctrl-63ab; KO, Sftpc-63ab. The median and quartiles are shown by dashed and dotted lines, respectively. The numbers of cells are shown at the bottom. *P < 0.05 and ***P < 0.001, by 1-way ANOVA with Tukey’s test. (**I**) Reacidification of LBs after removal of ATP in AT2 cells from Sftpc-63ab mice. Overlapped LysoTracker Green and FM4-64 fluorescence (orange, indicated by the arrow) suggests that the fusion pore was closed and luminal pH was reacidified. Scale bar: 5 μm. (**J** and **K**) Transmission electron microscopy images and cross-sectional areas (CSAs) of LBs from Ctrl-63ab and Sftpc-63ab mice. *n* = 41 and 94 LBs, respectively. Scale bars: 1 μm.

To investigate lung inflation–induced Ca^2+^ transients in AT2 cells deficient for TMEM63A/B, we delivered adeno-associated viruses (AAV) carrying Cre-dependent jGCaMP7s-mCherry into Sftpc-63ab mouse lungs via intratracheal instillation. jGCaMP7s is an optimized version of the GFP-based Ca^2+^ indicator (30), and mCherry is a red fluorescent protein serving as s Ca^2+^-insensitive reference. The Sftpc-CreERT2 mice were used as a control and instilled with the AAV in the same way. As indicated by the density of mCherry^+^ cells, the transduction efficiency was comparable between Sftpc-63ab and Sftpc-CreERT2 mice (Figure 5, A and B). However, in contrast to the evident inflation-induced Ca^2+^ transients in Sftpc-CreERT2 mouse lungs, no Ca^2+^ transients were observed in Sftpc-63ab lungs, neither in lobes that were still inflatable nor in fully collapsed lungs (Figure 5C and Supplemental Videos 12–14).

The absence of inflation-induced Ca^2+^ transients in AT2 cells would result from deficiency of mechanosensitive ATP release, hence, we hypothesized that a supply of exogenous ATP to alveoli would rescue the lethal phenotype of *Tmem63a*/*b-*cDKO. Unfortunately, daily inhalation of aerosolized ATP solution after tamoxifen induction did not significantly extend survival of the Sftpc-63ab and Ager-Sftpc-63ab mice (Figure 5D). Primary AT2 cells from Sftpc-63ab mice were able to respond to extracellularly applied ATP with Ca^2+^ transients (Figure 5E) and LB fusion to the plasma membrane, as indicated by staining for concentrated surfactant phospholipids with the FM4-64 dye that entered LBs through the fusion pore (Figure 5F). When ATP-treated AT2 cells from Ctrl-63ab mice were subjected to cyclic strain, some of the fused LBs lost FM4-64 fluorescence within 10 minutes (Figure 5F, upper panels), suggesting release of the phospholipid content. However, such a phenomenon was not observed in Sftpc-63ab AT2 cells treated in the same way (Figure 5F, lower panels). After cyclic strain for 2 hours, Ctrl-63ab cells lost most of the FM4-64^+^ prefused LBs, suggesting very high exocytic activity induced by cell stretch (Figure 5G). In contrast, the number of FM4-64^+^ LBs in Sftpc-63ab cells was only slightly reduced (Figure 5G), which indicates that surfactant from prefused LBs could not be efficiently released without TMEM63A/B, even in the presence of ATP and mechanical stretch. In AT2 cells without ATP treatment, cyclic strain for 2 hours significantly increased the number of FM4-64^+^ LBs in Ctrl-63ab cells, but had no effect on Sftpc-63ab cells (Figure 5H). A schematic description for these results is provided in Supplemental Figure 8.

When ATP was washed out and the cells were maintained in the static condition, some fused LBs became luminally reacidified within 20 minutes (indicated by the appearance of LysoTracker fluorescence overlapped with FM4-64 staining; Figure 5I), suggesting that the fusion pore could be closed if the exocytosis of fused LBs failed. Transmission electron microscopy showed more enlarged LBs with disordered internal membrane sheets in Sftpc-63ab AT2 cells than in control cells (Figure 5, J and K), which is consistent with the observation from LysoTracker staining in live AT2 cells from acute lung slices (Supplemental Figure 7A) and probably caused by abnormal contact and fusion between LBs that could not be exocytosed in the absence of TMEM63A/B.

### TMEM63A/B are essential for mechanosensing in AECs.

**Figure 6:**
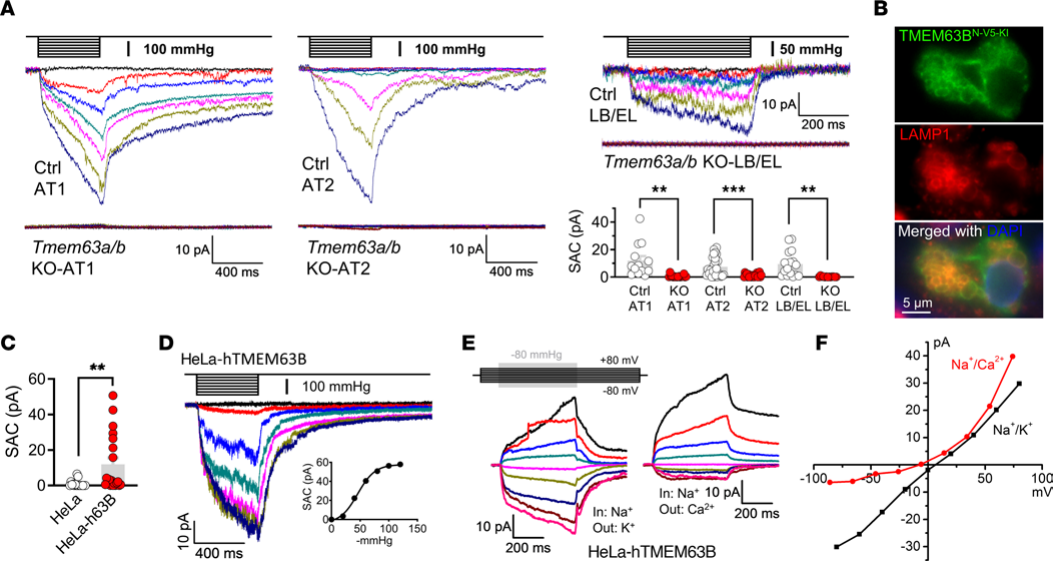
TMEM63A/B are essential for mechanosensitive currents in AECs. (**A**) SACs were abolished in AT1 cells, AT2 cells, and LB/EL from *Tmem63a*/*b-*cDKO mice. Current amplitudes at –80 mV and –80 mmHg for AT1 and AT2 cells and –60 mV and –60 mmHg for LB/EL were used for comparison. *n* = 13 (Ctrl-AT1, from Ctrl-63ab mice); *n* = 18 (KO-AT1, from Ager-63ab); *n* = 25 (Ctrl-AT2, from Ctrl-63ab); *n* = 21 (KO-AT2, from Sftpc-63ab); *n* = 16 (Ctrl-LB/EL, from Ctrl-63ab); *n* = 13 (KO-LB/EL, from Sftpc-63ab) cells. **P < 0.01 and ***P < 0.001, by 2-tailed, unpaired Student’s *t* test. (**B**) Immunofluorescence images of endogenous TMEM63B with N-terminal 2×V5-tag–knockin (N-V5-KI) in an AT2 cell in a frozen section of mouse lung. LAMP1 is a marker of LBs. Scale bar: 5 μm. (**C**) Amplitudes of SACs in control and human TMEM63B-transfected (h63B-transfected) HeLa cells. Holding potential: –80 mV; pressure: –80 mmHg. *n* = 18 and 20 for HeLa and HeLa-h63B cells, respectively. **P < 0.01, by 2-tailed, unpaired Student’s *t* test. (**D**) Representative traces of SACs under the cell-attached configuration in a h63B-transfected HeLa cell. The pressure-current relationship is shown in the inset. (**E** and **F**) SAC traces and current-voltage relationships of inside-out recordings from h63B-transfected HeLa cells suggesting selectivity for Na^+^ and K^+^ over Ca^2+^.

To further explore the role of TMEM63A/B in the mechanotransduction in AECs, we examined the mechanosensitive currents in AT1/AT2 cells and LB/EL vesicles from *Tmem63a*/*b-*cDKO mice. Pressure clamp under the cell-attached mode detected only negligible stretch-activated currents in AT1 and AT2 cells from-cDKO mice, while the mechanosensitive currents were totally abolished in the LB/EL vesicles from TMEM63A/B-deficient AT2 cells (Figure 6A).

Given the lack of reliable antibodies against endogenous TMEM63A/B, we were unable to directly detect their localization in AT1 and AT2 cells with immunofluorescence. Instead, we performed genome editing in mouse lungs with CRISPR/Cas9 technology to knock in the 2×V5-tag sequence immediately after the start codon of the *Tmem63b* gene. Immunofluorescence against the V5 tag showed that TMEM63B was mainly localized at the limiting membrane of LBs in AT2 cells, as indicated by the marker LAMP1 (Figure 6B). No obvious immunofluorescence was detected at the plasma membrane of AT1 and AT2 cells, probably because the density of TMEM63B proteins at the cell surface is much lower than that of LBs. Fusion of LBs with the plasma membrane may allow diffusion of a small amount of TMEM63B from LBs to the cell surface.

Overexpression of fluorescent protein–tagged TMEM63A/B in HeLa cells and subsequent treatment with vacuolin-1 revealed that both TMEM63A and TMEM63B were present at endolysosomal membranes but did not completely overlap, whereas TMEM63B was additionally localized at the plasma membrane (Supplemental Figure 9). Cotransfection with the early endosome marker RAB5A and staining with LysoTracker Green, an indicator of acidic lysosomes and late endosomes, suggested the preference of TMEM63B for early endosomes and the universal presence of TMEM63A in early and late endosomes and lysosomes (Supplemental Figure 10). We further confirmed the presence of TMEM63B at the plasma membrane by nonpermeabilized immunofluorescence against a V5 tag fused at the N-terminus of TMEM63B (V5-TMEM63B), which is located extracellularly (Supplemental Figure 11A). The same strategy produced very dim staining against Myc-TMEM63A, suggesting that TMEM63A is rarely trafficked to the plasma membrane (Supplemental Figure 11A).

Large stretch-activated currents were recorded in 7 of 20 HeLa cells transfected with TMEM63B (Figure 6C) and exhibited the same properties as the currents in AECs, including slow activation, no inactivation, and higher permeability for Na^+^ and K^+^ than for Ca^2+^ (Figure 6, D–F). This suggests that TMEM63B could recapitulate the properties of the mechanosensitive channel in AECs. Although Ca^2+^ permeability of TMEM63B has been proposed by a previous study based on hypotonic stimulation (18), we suppose this would be questionable because of some technical issues we reviewed recently (31), and, more important, we did not record convincing hypotonicity-induced currents in TMEM63B-transfected LRRC8A-KO HEK293 cells without VRAC activity (Supplemental Figure 3E).

As for the low ratio of successful recordings for large stretch-activated currents in HeLa cells, we found that TMEM63B was not evenly distributed but rather formed clusters at the plasma membrane, with a mean density of approximately 1 cluster/μm^2^ (Supplemental Figure 11B). The area of the micropipette tip in our experiment was approximately 1 μm^2^, which could only accommodate one TMEM63B cluster on average, therefore negative recordings could not be avoided.

Consistent with a previous report on TMEM63-transfected HEK293 cells (17), whole-cell recordings in TMEM63B-transfected HeLa cells did not show any cell indentation–induced currents. In TMEM63A/B-transfected HeLa cells, the vacuolin-1–induced vesicles were too small to perform patch-clamp recordings (Supplemental Figure 9). This implies that TMEM63A/B may function in HeLa cells to prevent the fusion of endolysosomes (ELs), however, their ion channel activity cannot be directly assessed because of technical limitations.

### Human TMEM63A/B rescued the lethal phenotype of Tmem63a/b-cDKO mice.

**Figure 7:**
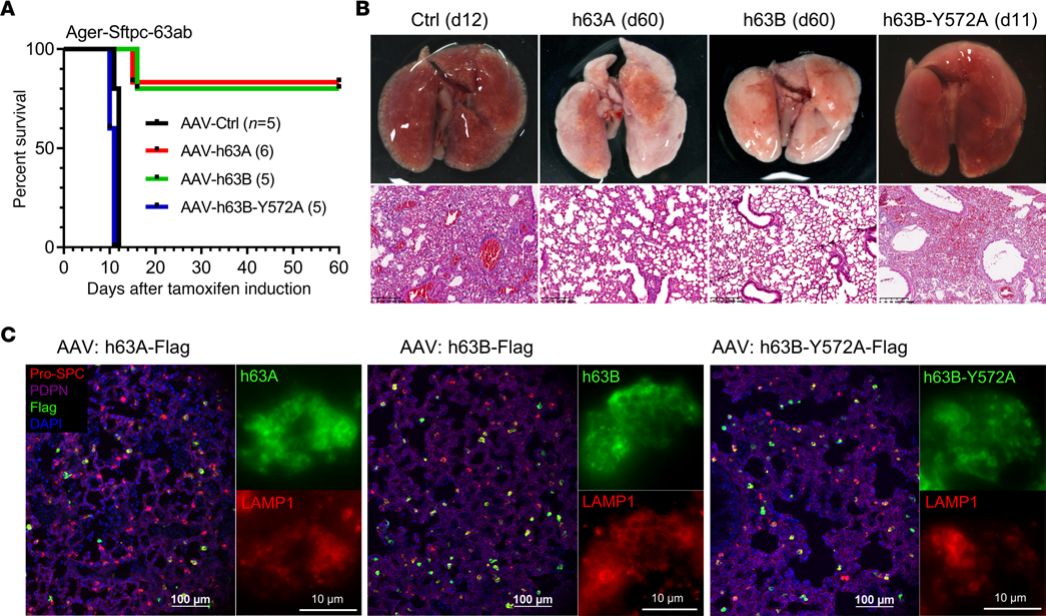
Human TMEM63A/B rescues respiratory failure in *Tmem63a*/*b-*cDKO mice. (**A**) Survival curves for *Ager-Sftpc-63ab*-cDKO mice that received intratracheally delivered AAVs with empty vector (Ctrl), human TMEM63A (h63A), human TMEM63B (h63B), or the h63B-Y572A mutant after tamoxifen induction. The numbers of mice are shown in parentheses. (**B**) Images show the relatively normal appearance and structure of h63A- and h63B-rescued lungs compared with the lungs transduced with control or h63B-Y572A AAV. Scale bars: 200 μm. d11, day 11; d12, day 12; d60, day 60. (**C**) Expression of h63A, h63B, and h63B-Y572A in mouse lungs and localization in AT2 cells. Images show immunofluorescence staining for Pro-SPC (AT2), PDPN (AT1), Flag (h63A/B), and LAMP1 (LBs). Scale bars: 100 μm and 10 μm.

We delivered AAVs encoding human TMEM63A (h63A) and TMEM63B (h63B) into the lungs of Ager-Sftpc-63ab mice by intratracheal instillation on the first day of tamoxifen injection. Both h63A and h63B successfully rescued the mice from respiratory failure, with 5 of 6 mice that received h63A and 4 of 5 mice that received h63B surviving for 2 months after tamoxifen induction (Figure 7A), before they were sacrificed for sampling. The lungs from these mice showed normal morphology and structure, with no signs of atelectasis or edema (Figure 7B). In contrast, a mechanoinsensitive pore mutant of h63B (Y572A, Supplemental Figure 12) failed to compensate for the loss of endogenous TMEM63A/B in cDKO mice (Figure 7, A and B). The transduction efficiency of h63A, h63B, and h63B-Y572A AAVs was comparable, as all had high expression in AT2 cells and prominent localization at the limiting membrane of LBs (Figure 7C). These results demonstrate that the mechanosensitive channel activity of TMEM63A/B was crucial for normal lung function, and this role of TMEM63A/B was conserved between mouse and human.

## Discussion

In the present study, we demonstrate that TMEM63A/B channels essentially supported alveolar expansion and breathing in both neonatal and adult mice by enabling sufficient surfactant secretion. Although the mechanism that triggers the first breath at birth is complicated (32, 33), pulmonary surfactant plays an essential role in this process by reducing the surface tension of the alveolar lining liquid, i.e., the resistance to breathing in the air. Preterm infants often develop neonatal respiratory distress syndrome due to surfactant deficiency (1). Little is known about the regulation of surfactant secretion in developing lungs at the prenatal stage. Mechanical force generated from inhalation of a small amount of amniotic fluid by fetal breathing movements before birth is essential for the differentiation of AT1 cells in lung development (34), which we suppose would also stimulate surfactant secretion in the fetal lung. We detected stretch-activated currents in E17.5 mouse AT2 cells (Supplemental Figure 3F), with the same properties as TMEM63 currents in adult AT2 cells. Therefore, the deficiency of TMEM63A/B channels may have diminished alveolar surfactant levels and caused the failure to breathe after birth via the same mechanism we showed in adult lungs.

Surfactant dysfunction is a characteristic of acute respiratory distress syndrome (ARDS) in adult patients (35). Surfactant activity is compromised in ARDS because of a series of pathological changes, such as inflammatory damage of AT2 cells, reduced surfactant synthesis and secretion, altered surfactant composition, accelerated surfactant breakdown, and functional inhibition by plasma components (36). As a consequence, alveolar surface tension is increased, which leads to poor alveolar compliance, alveolar collapse, and severe hypoxemia. Despite these findings, exogenous surfactant therapy only improved oxygenation in some patients with ARDS but did not confer a survival benefit (37, 38). This is generally attributed to the heterogeneity of ARDS and the lack of an optimal therapeutic strategy, including compositions and doses of surfactant and the timing and methods of surfactant delivery (36, 39). Further studies on the pathophysiological mechanisms of ARDS and surfactant dysfunction are required to improve the efficacy of surfactant therapy. It will be interesting to investigate whether the expression and activity of TMEM63A/B channels are impaired in ARDS and to determine their potential as therapeutic targets.

It should be noted that, although we have found that the concentrations of SPC and DPPC, 2 main components of pulmonary surfactant, were dramatically reduced in the BALF of *Tmem63a*/*b-*cDKO mice, the surface tension of the BALF needs to be measured directly using a pulsating bubble surfactometer or a Wilhelmy balance (40) to provide definitive evidence for surfactant deficiency, which is a limitation of our study.

The mechanisms of mechanosensitive ATP release have been extensively studied in various cell types, with 3 possible pathways suggested: ATP-conducting channels, vesicular exocytosis, and membrane leakage (3). Channels proposed to permeate ATP include gap junctional hemichannels (connexins and pannexins), calcium homeostasis modulator 1 (CALHM1) channels, VRACs, and Maxi Cl^–^ channels (41). Connexins, pannexins, and CALHM1 are large-pore channels that are nonselective for cations, anions, and small molecules (42). If these channels are opened by membrane stretch to allow ATP permeation, they should also permeate small ions such as Ca^2+^, Na^+^, K^+^, and Cl^–^. As we did not observe stretch-activated Ca^2+^ influx into AT1 and AT2 cells, these channels are unlikely to conduct mechanosensitive ATP release from AECs. In agreement with this, blockers of connexins and pannexins did not inhibit lung inflation–induced, ATP-mediated Ca^2+^ transients in AT2 cells. As for CALHM1, its expression was not detected in AT1 or AT2 cells by single-cell RNA-Seq (Mouse Cell Atlas and Human Protein Atlas). Similar to these nonselective channels, ATP leakage due to stretch-induced membrane injury should also be accompanied by immediate and massive Ca^2+^ influx (3), which was not observed in our experiment.

VRACs and maxi-Cl^–^ channels are recognized as anion-selective channels, and thus they may conduct negatively charged ATP with no or very weak cation permeation (43). Nevertheless, we found that blockers of these channels did not affect lung inflation–induced, ATP-mediated Ca^2+^ transients in AT2 cells. Moreover, the patch-clamp recordings indicated that the stretch-activated channel in AT2 cells was highly permeable to K^+^ and Na^+^ and that the single-channel conductance was too small to resolve, which are apparently not properties of VRACs or maxi-Cl^–^ channels. Therefore, these 2 types of anion channels are unlikely to play an important role in mechanosensitive ATP release from AT2 cells.

By blocking ATP transport into LBs with clodronate, we demonstrate that exocytosis from LBs was probably the major pathway of lung inflation–induced ATP secretion, which is consistent with previous findings that ATP is enriched in LBs (5) and released independently of ATP-conducting channels (7). It needs to be mentioned that the membrane permeability of clodronate is very low and is normally packed into liposomes for intracellular delivery (44). However, as a bisphosphonate, clodronate may be transported into AT2 cells by the sodium-phosphate cotransporter SLC34A2, which is abundantly expressed in AT2 cells (45). In addition, bisphosphonate can be endocytosed into ELs and then released into cytosol via the SLC37A3-ATRAID transporter complex in osteoclasts (46). The same pathway may also exist in AT2 cells, since SLC37A3 and ATRAID are ubiquitously expressed.

Previous studies have described the effects of ATP, Ca^2+^, and cell stretch on surfactant secretion (4), however, some of the mechanistic steps are still unclear. Stimulation with ATP efficiently promotes LB fusion to the plasma membrane, but its effect on subsequent surfactant release is limited and can be substantially enhanced by cell stretch (8). In line with this, the expansion of the fusion pore has been demonstrated to be a slow and discontinuous process in the static condition (47). This raised the question of how cell stretch promotes surfactant release from fused LBs.

Unlike other exocytic materials such as neurotransmitters, pulmonary surfactant is a bulky and hydrophobic lipoprotein complex that cannot be released into extracellular fluid simply by diffusion through the fusion pore (48). After LB fusion to the plasma membrane, surfactant is still trapped in LBs, and spontaneous expansion of the fusion pore occurs slowly (47). The constrictive fusion pore therefore acts as a mechanical barrier obstructing surfactant release (8). Moreover, even when the fusion pore is dilated, surfactant does not readily diffuse out of fused LBs (48). A mechanical force that compresses LBs is required to squeeze the surfactant out in an “all-or-none” manner (8). It has been found that the formation and contraction of actin coats on fused LBs are essential for surfactant secretion (48, 49). Contraction of actin coats was visualized with GFP-tagged actin overexpressed in ATP-treated AT2 cells in the static condition (48), which, to our knowledge, is the only observation for the shrinking process of fused LBs so far. However, it is unclear whether the contraction of actin coats is an active process that applies force on LBs or a passive reassembling process associated with shrinking of LBs to prevent backward enlargement, as the ATP-activated cation channel P2X4 at the limiting membrane of LBs also plays a role in fusion pore expansion and surfactant release (5, 50).

On the basis of our findings that TMEM63A/B-deficient AT2 cells failed to release surfactant in response to cell stretch, we propose a TMEM63A/B-mediated mechanism for this process: when AT2 cells are stretched during inspiration, LBs are also mechanically stressed due to their large size and association with the cytoskeleton (51). Then, TMEM63A/B at the limiting membrane of LBs are activated due to increased membrane tension. For LBs fused on the plasma membrane, stretch-activated Na^+^ flux from the LB lumen to the cytosol through TMEM63A/B can theoretically generate an osmotic force that shrinks fused LBs and squeezes surfactant out to the cell surface, like the mode demonstrated in neuroendocrine cells (52). Additionally, as TMEM63A/B activity is also detected at the plasma membrane, the osmotic gradient can potentially cause water influx and transient cell swelling, which may help to expand the fusion pore. Because ATP is coreleased with surfactant from fused LBs (5), this hypothesis also explains how mechanosensitive ATP release is conducted. Unfortunately, since it is currently a technical challenge to visualize the size of LBs and surfactant release during cell stretch, which requires very high resolution, a wide field of view (due to cell movement), perfect focus, and ultrafast imaging, we were unable to obtain direct evidence for the shrinkage of fused LBs during cell stretch.

For LBs, the stretch-activated currents measured at –60 mmHg and –60 mV were similar to (mouse) or even larger (human) than those of the plasma membrane at –80 mmHg and –80 mV (Figure 3, B and F), suggesting that LBs are more sensitive to membrane stretch, which further supports our hypothesis of osmotic force–driven exocytosis of LBs. As for LBs not fused with the plasma membrane (with intact limiting membrane), activation of TMEM63A/B may also cause osmotic shrinkage, but the size of LBs could be quickly recovered by counteracting ion flux via other channels and transporters after stretch, without loss of luminal contents.

In the patch-clamp experiment, we found that cell indentation was not a proper way to activate TMEM63A/B, and very high vacuum pressure was required to induce robust stretch-activated currents in the cell-attached recording. It needs to be explained that the vacuum pressure used in the pressure clamp cannot be directly compared with the air pressure that drives alveolar expansion. We calculated negative pressure-induced membrane area changes in the micropipette under the cell-attached configuration using published data (53). Compared with the membrane area at 0 mmHg, –100 mmHg pressure only increased the membrane area by approximately 85% (Supplemental Figure 13). In contrast, the surface area of AECs can expand by several fold during breathing (54, 55), with a transpulmonary pressure (alveolar pressure minus intrapleural pressure) of less than 10 mmHg as the driving force (56). Therefore, although very high pressure was used in our experiment, the extent of membrane stretch was still within the physiological range. Lateral stretch of AECs in vivo should be much more efficient in increasing membrane tension and activating mechanosensitive channels.

In lung mechanobiology, the Piezo2 channel has been recognized as the stretch sensor in sensory neurons innervating the airway, which limits overinflation of the lung by triggering the Hering-Breuer inspiratory reflex (57). It will be interesting to determine whether lung inflation–induced TMEM63 channel activity contributes to mechanotransduction in sensory neurons, either through ATP signaling or direct activation in neurons. As genetic variants of *TMEM63A/B/C* have been identified in patients with neurodevelopmental disorders (58–60), further studies are required to understand the functions of TMEM63 channels in the nervous system. In humans, *TMEM63A* is highly expressed in oligodendrocytes, and *TMEM63B/C* expression is present in both neurons and glial cells (31). Similarly, *Tmem63a* expression is absent, while expression of *Tmem63b/c* is detected in most mechanosensory neurons in mouse dorsal root ganglion (61). The function of TMEM63 channels is critically dependent on their subcellular localization, which is completely unknown in neurons and glial cells at the moment. Considering the broad expression of TMEM63A/B (31), studies of their roles in other mechanically active organs, such as the heart, gut, and bladder, are also expected.

In summary, we found that TMEM63A/B mechanosensitive channels in AT2 cells controlled the release of pulmonary surfactant and ATP by sensing and transducing mechanical force on LBs during alveolar expansion. Deficiency of TMEM63A/B resulted in surfactant insufficiency in alveoli, which subsequently caused atelectasis and respiratory failure. The exact mechanism of TMEM63A/B in promoting surfactant release from LBs and their role in AT1 cells need to be further investigated.

## Methods

### Mice.

KO or transgenic mouse strains were purchased from the qualified providers listed in Supplemental Table 2. To obtain constitutive *Tmem63a/b* double-KO mice, *Tmem63a^–/–^* *Tmem63b^+/–^* mice were first generated by crossing *Tmem63a^+/–^* and *Tmem63b^+/–^* mice for 2 generations and then using them to breed *Tmem63a^–/–^* *Tmem63b^–/–^* mice. For *Tmem63a/b*-cDKO, *Tmem63a^fl/fl^* *Tmem63b^fl/fl^* mice were first generated and then crossed with Cre mice for 2–3 generations to obtain Cre^+^ *Tmem63a^fl/fl^* *Tmem63b^fl/fl^* mice.

The mice were maintained at the Experimental Animal Center of Southwest Medical University under specific pathogen–free (SPF) conditions with standard 12-hour light/12-hour dark cycles and free access to food and water. Mice of both sexes at different ages were used in the experiments.

### Cell line.

HeLa cells were purchased from the American Type Culture Collection (ATCC) and cultured in DMEM/F-12 medium with 10% FBS, 100 units/mL penicillin, and 100 μg/mL streptomycin in a 5% CO_2_ incubator at 37°C (abbreviated as “normal culture condition” hereafter). The cells were tested negative for mycoplasma contamination and were transfected with plasmids using Lipofectamine 2000 (2 μL/μg DNA), followed by plating on glass coverslips for fluorescence imaging or patch-clamp experiments.

### Ca^2+^ imaging in ex vivo lungs.

GCaMP-expressing mice were sacrificed with CO_2_ inhalation and subjected to endotracheal intubation with a blunted syringe needle (tracheal catheter). The lung, heart, and intact trachea were carefully dissected together from the mice. The trachea was tightly tied onto the tracheal catheter with surgical sutures during dissection. The lung was placed on a glass coverslip on the microscope, and the tracheal catheter was connected to a ventilator. The frequency of ventilation was 1 Hz in all experiments. Fluorescence imaging of GCaMP, tdTomato, and mCherry was conducted with FITC, TRITC, and Texas Red filter sets, respectively. The focus was adjusted before and after mechanical ventilation for best-quality imaging. The Nikon Eclipse Ti inverted microscope, the ORCA-Flash4.0 sCMOS camera (Hamamatsu, Japan), and NIS-Elements software (Nikon, Japan) were used for fluorescence imaging.

For chemical blockers used in the lungs, we used 2- to 3-fold higher concentrations than are normally used for cultured cells, since the compounds could be diluted by alveolar fluid during mechanical ventilation. Chemicals were diluted in 100 μL physiological extracellular solution (140 mM NaCl, 5 mM KCl, 1 mM MgCl_2_, 2 mM CaCl_2_, 10 mM HEPES, and 10 mM glucose, pH7.4) and instilled into the lung via the tracheal catheter.

### Isolation and culturing of AT2 cells.

After euthanasia with CO_2_, the lungs and hearts of mice were surgically exposed. Sterile 0.9% NaCl was perfused from the right heart apex until the heart was visually bleached. Then, endotracheal intubation was performed, and 2 mL dispase solution (1 mg/mL dissolved in PBS, MilliporeSigma) was slowly infused into the lung, and left for 5 minutes. Immediately after injection of 0.5 mL low-melting-point agarose (1%, dissolved in PBS), the lung was covered with ice to solidify the agarose over a 2-minute period. The lung was removed from the mouse and digested in dispase solution for 45 minutes at room temperature on a shaker, and then transferred to 7 mL 0.01% DNase I (dissolved in DMEM/F-12) and cut into small pieces using fine scissors. The cell suspension was collected for filtration with a 100 μm cell strainer and centrifuged at 124*g* for 4 minutes at 4°C. Next, the cell suspension was cultured in a normal Petri dish under normal culture conditions. After 12 hours, unattached cells (mainly AT2 cells) were collected by centrifugation at 124*g* for 4 minutes at 4°C and then plated on glass coverslips or silicon membranes (0.1 mm thickness) precoated with fibronectin (50 μg/mL overnight) under normal culture conditions.

Human AT2 cells were isolated from lung tissues according a previously published protocol (62) with minor modifications. Briefly, the samples were instilled with PBS to remove blood and digested with 0.25% trypsin for 45 minutes at 37°C. The tissue was then cut into fine pieces in the presence of FBS and treated with DNase I. The cells were filtered with a 100 μm cell strainer, and the same steps as described for the mouse AT2 cells above were followed.

### Preparation of lung slices.

Endotracheal intubation was performed on euthanized mice. The lung was infused with 2 mL 1.5% low-melting-point agarose solution (58.2 mM NaCl, 2.7 mM KCl, 0.9 mM CaCl_2_, 0.4 mM MgSO_4_, 0.6 mM NaH_2_PO_4_, 12.6 mM HEPES, 13 mM NaHCO_3_, and 8.4 mM glucose, pH7.2), and the lung was covered with crushed ice for 20 minutes. Then, the lung was removed from the mouse and cut into slices of 250 μm thickness in 4°C cold slicing solution (116.4 mM NaCl, 5.4 mM KCl, 1.8 mM CaCl_2_, 0.8 mM MgSO_4_, 1.2 mM NaH_2_PO_4_, 25.2 mM HEPES, 26.1 mM NaHCO_3_, and 16.7 mM glucose, pH7.2) using a vibratome (Leica VT 1000S). The lung slices were stored in slicing medium at 4°C for fluorescence imaging or electrophysiological experiments.

### Electrophysiology.

Mouse and human AT2 cells were characterized from crude isolated cells by LysoTracker staining on LBs, which are much larger and brighter than lysosomes in other cell types. All recordings were performed at room temperature (~25°C).

Macroscopic stretch-activated currents of AT2 and transfected cells were recorded under the cell-attached or inside-out configuration. Resistances of the micropipettes were 3–4 MΩ. Membrane patches were stretched by 500 ms negative pressure pulses through the glass micropipette. The negative pressures were generated by the high-speed pressure control device (HSPC-2, ALA-Scientific) with a –20 mmHg increase for each sweep and an intersweep duration of 10 seconds. Signals were sampled at 10 kHz and filtered at 3 kHz.

For AT1 cells with *Tmem63a/b*-cDKO (Ager-63ab), AAVs carrying CMV-DIO-EGFP were instilled into the lung to label Cre^+^ cells with ablated *Tmem63a/b*. The AT1-attached pressure clamp was conducted in acute lung slices in physiological extracellular solution. Resistances of the micropipettes were 3–4 MΩ. Only EGFP^+^ AT1 cells were considered authentic *Tmem63a/b*-cDKO cells because Ager-CreERT2 does not induce DNA recombination in all AT1 cells.

To record mechanosensitive currents in LB/EL vesicles, AT2 cells were treated with 2.5 μM vacuolin-1 in culture medium overnight. Enlarged LB/EL vesicles were isolated and patch clamped under vesicle-attached configuration. Both the pipette and bath solutions for LB/EL vesicles were physiological intracellular solutions (135 mM KCl, 10 mM NaCl, 0.5 mM MgCl_2_, 3.8 mM CaCl_2_, 10 mM HEPES, and 10 mM EGTA, pH7.2). Resistances of the micropipettes were 5–7 MΩ. Pressure clamp with a –10 mmHg increase for each sweep and an intersweep duration of 10 seconds was conducted.

For ion selectivity assays, solutions including 150 mM NaCl plus 10 mM HEPES, 150 mM KCl plus 10 mM HEPES, 100 mM CaCl_2_ plus 10 mM HEPES, 100 mM MgCl_2_ plus 10 mM HEPES, and 150 mM NMDG-Cl plus 10 mM HEPES (pH 7.4 for all) were used for pressure clamp under the cell-attached or inside-out configuration. Voltage steps from –100 to +100 mV or –80 to +80 mV with a 20 mV increase and –80 mmHg pressure were applied. Liquid junction potentials were calculated in Clampex and corrected after experiments. Relative permeability to Na^+^ was calculated with the Goldman-Hodgkin-Katz Equation detailed previously (63).

### Tamoxifen administration.

Tamoxifen was dissolved in corn oil to 20 mg/mL by sonification and kept in the dark. Both the CreERT2^+^ and control mice were intraperitoneally injected with tamoxifen solution (75 mg/kg body weight per day) for 5 consecutive days.

### Micro-CT.

Micro-CT for isoflurane-anesthetized mice was performed using the Siemens Inveon Multi-Modality System (Siemens Healthineers), with the following sampling parameters: 80 kV voltage, 500 μA current, 200 ms exposure, 220° rotation, 120 projections, and an 8.8 × 13.29 cm field of view. Acquisitions were reconstructed with Feldkamp’s algorithm using software provided with the system. Computed intensities from representative areas of approximately 40 mm^3^ volume in left and right lungs were averaged as the mean lung volume intensity.

### Saturation of SpO_2_ monitoring.

Mice were anesthetized with isoflurane, and the hair was removed bilaterally from the neck with depilatory. A small collar clip with a pulse oximeter sensor (MouseOx Plus, Starr Life Sciences) was placed on the neck to monitor the SpO_2_. Data were recorded after the mice were returned to their home cages and completely recovered from anesthetization.

### BAL.

Immediately after euthanasia, the mouse trachea was exposed and intubated. PBS (1 mL) was gently infused into the lung through a catheter followed by aspiration with a syringe. This procedure was repeated 5 times and BALF (0.7–0.9 mL) was collected into a tube and placed on ice. After centrifugation at 400*g* for 5 minutes at 4°C, the supernatant was either immediately used or stored at –80°C.

### Immunofluorescence and H&E staining.

Lung tissues were fixed with 4% paraformaldehyde, dehydrated in 20%–30% sucrose, frozen or paraffin embedded, and then sectioned. Immunofluorescence on frozen or paraffin-embedded sections and HE staining on paraffin sections were performed using standard protocols (64). Details on the antibodies used are provided in Supplemental Table 2.

For cell-surface immunofluorescence, HeLa cells transfected with Myc-TMEM63A and V5-TMEM63B plasmids were washed 3 times with PBS, incubated with rat anti–Myc tag and rabbit anti–V5 tag primary antibodies diluted 1:500 with cold culture medium at 4°C for 0.5 hours, and then washed 3 times with cold PBS and fixed with 2% paraformaldehyde. Fixed cells were blocked with 20% skim milk, incubated with AF647-conjugated donkey anti–rat IgG and AF488-conjugated donkey anti–rabbit IgG secondary antibodies (1:200) at room temperature for 1 hour, washed 3 times with PBS, and then mounted for imaging. For immunofluorescence of permeabilized cells, cells were fixed with 2% paraformaldehyde, permeabilized with chilled methanol, blocked with 20% skim milk, and then incubated with primary and secondary antibodies.

### ELISA of surfactant protein C and ATP measurement.

The mouse SPC ELISA Kit (ab252366, Abcam) and the ATP Determination Kit (S0026, Beyotime) were used to detect the concentrations of mature surfactant protein C (SPC) and ATP in BALF from the mice, respectively. The procedures were carried out according to the manufacturers’ instructions.

### Determination of DPPC.

Standard DPPC was purchased from Avanti Polar Lipids (catalog 850355). HPLC-grade methanol, chloroform, 2-isopropanol, and acetonitrile were purchased from MilliporeSigma, and ammonium acetate was purchased from Mallinckrodt Baker.

The lavage fluid was directly used in liquid chromatography/tandem mass spectrometry (LC-MS/MS) (QTRAP 5500+, AB Sciex) for DPPC determination. LC separation was performed on a Kinetex C18 column (2.6 μm, 100 × 2.1 mm 100 Å, Phenomenex) at a flow rate of 0.45 mL/min and a column temperature of 40°C with a loading volume of 2 μL. The mobile phase consisted of mobile phase A (water/methanol/acetonitrile = 1:1:1; 7 mM ammonium acetate) and mobile phase B (2-isopropanol, 7 mM ammonium acetate). The eluted condition was as follows: 0–0.8 minutes, 50% B; 0.8–4.80 minutes, 50%–80% B; 4.80–4.90 minutes, 80%–98% B; 4.90–5.50 minutes, 98%–50% B; 5.50–6.30 minutes, 50% B. DPPC, eluted at 2.84 minutes, and quantification were carried out in multiple reaction monitoring (MRM) mode, with the *m/z* at 734.6 (Q1) and 184.3 (Q3) in positive mode throughout LC-MS/MS. Declustering potential (DP) and collision energy (CE) values were 100 and 35, respectively.

### Intratracheal instillation of AAV into mouse lungs.

The mouse was anesthetized with isoflurane, and an endotracheal cannula was inserted into the trachea. AAV was infused into the lung using a manually pulled long and thin micropipette tip through the endotracheal cannula. Mechanical ventilation was performed for 5 minutes after instillation. Anesthetization was terminated when the mouse recovered spontaneous breathing at a normal rhythm within approximately 10 minutes, after which the mouse was returned to its home cage.

An AAV6-based triple-mutant capsid (AAV6.2FF) (65) was used to deliver transgenes into mouse lungs. The AAV dosage/mouse was 50 μL CMV-h63B-3xFlag-tWPA (2.27 × 10^12^ vg/mL), 25 μL CMV-h63A-3xFlag-tWPA (9.26 × 10^12^ vg/mL), 25 μL CMV-MCS(Ctrl)-3xFlag-tWPA (8.95 × 10^12^ vg/mL), 50 μL CMV-h63B-Y572A-3xFlag-tWPA (3.34×10^12^ vg/mL), and 25 μL CAG-DIO-jGCaMP7s-mCherry-WPRE (6.23 × 10^12^ vg/mL). Instillation of the AAVs was performed 12–16 hours after the first injection of tamoxifen.

### Genome editing in mouse lungs.

The AAV U6-spgRNA(Tmem63b)-donor(V5tag)-WPRE, which carries the gRNA sequence GGCCAGCAAGAACGGCAGCA that targets the start codon region of the murine *Tmem63b* gene and the 2×V5 tag sequence with 400 bp homology arms on both sides, was instilled into the lungs of *SpCas9*-transgenic mice (50 μL/mouse, 9.06 × 10^12^ vg/mL). After 3 weeks, the lungs were dissected and frozen sectioned for immunofluorescence.

### ATP/saline inhalation.

Ager-Sftpc-63ab, Sftpc-63ab, and Ctrl-63ab mice were injected with tamoxifen for 5 consecutive days. On the day after the last tamoxifen injection, the mice were placed in an anesthesia induction chamber, in which aerosolized Na_2_ATP solution (200 mM) or saline was provided by connecting to a nebulizer. Aerosol inhalation was conducted for 30 minutes each time, 3 times per day, until the cDKO mice died.

### Mechanical strain of AT2 cells.

To examine the Ca^2+^ response directly induced by mechanical strain, AT2 cells were grown on a fibronectin-coated silicone membrane (~12 mm in diameter, 0.1 mm thickness, transparent) firmly adhered to the glass of a glass-bottomed dish. The cells were stained with 5 μM Fluo-4 AM in culture medium for 20 minutes, which was then changed to a physiological extracellular solution for Ca^2+^ imaging. A glass micropipette was moved with a micromanipulator toward the cell in the same way for the patch-clamp procedure. The tip of the micropipette was then pierced into the silicone membrane close to the cell. Time-lapse imaging for fluo-4 fluorescence was started with an exposure duration of 100 ms and no intervals. The micropipette tip was moved horizontally 20 μm away from the cell and returned to its original position during imaging. The fluorescence intensity of each cell was measured from images at different time points.

For the strain-induced surfactant release experiment, a custom strain chamber with a silicone membrane (10 cm × 10 cm, 0.1 mm thickness) stretched by cyclic vacuum pressure from an electric breast pump was used. AT2 cells were grown on the center of a fibronectin-coated silicone membrane and subjected to cyclic strain or static culture. The silicone membrane was then removed from the chamber and mounted onto the microscope to capture high-resolution images of AT2 cells for LB quantification.

To observe LBs in the same AT2 cell before and after strain, the silicone membrane was directly mounted onto the ×100 objective after ATP treatment and FM4-64 staining. After capturing an image for an individual cell, a blunted glass micropipette driven by a micromanipulator was used to stretch the cell by pressing its adjacent silicone membrane area with an angle of approximately 45^°^, a distance of 200 μm, and a frequency of 1 Hz. After strain for 5 minutes and 10 minutes, the cell was refocused and imaged if it was not detached.

### Transmission electron microscopy.

Transmission electron microscopy was performed at the Lilai Biomedical Experimental Center (Chengdu, China). Fresh lung samples of approximately 1 mm thickness were prefixed with 3% glutaraldehyde and then postfixed in 1% osmium tetroxide, dehydrated in series acetone, infiltrated in Epox 812, and embedded. The semithin sections were stained with methylene blue. Ultrathin sections were produced by cutting with a diamond knife and were then stained with uranyl acetate and lead citrate. The sections were examined with a JEM-1400-FLASH Transmission Electron Microscope (JEOL).

### Statistics.

Data are presented as the mean ± SEM unless otherwise indicated. In the violin plots, the median and quartiles are shown. A 2-tailed Student’s *t* test was used in experiments with only 2 groups. One-way ANOVA with Tukey’s test was used to identify statistical differences among 3 or more groups. Two-way ANOVA with Šidák’s test was used when the response was affected by 2 factors (time and drug treatment). A *P* value of less than 0.05 was considered statistically significant.

### Study approval.

All animal experimental procedures were reviewed and approved by the ethics committee of Southwest Medical University and in accordance with national guidelines for housing and use of laboratory animals. Human AT2 cells were isolated from healthy lung tissues cut off from tumors from 2 patients (*n* = 1 male and 1 female) undergoing lung cancer surgery. Isolated cells were used for Ca^2+^ imaging and patch clamp experiments. The experiment using human lung samples was approved by the ethics committee of Southwest Medical University. Informed consent was obtained from all participants.

### Data availability.

Data generated in this study are available in the Supplemental Supporting Data Values file or from the corresponding author upon reasonable request.

## Author contributions

Study design, supervision, and manuscript preparation: GLC and BZ; histology and microscopic imaging: JY Li, JWL, QZ, JY Liu, and BZ; patch clamp experiments: GLC, JY Li, XC, JY Liu, JW, and NW; animal experiments: GLC, JY Li, QZ, JY Liu, JW, JPW, LY, and YPL; molecular cloning and mutagenesis: JWL and JJL; primary cell culturing: XC, ML, and HQY; genome editing: GLC and BZ; LC-MS/MS: ZH and JD; structural modeling: JZ. The order of the co–first authors’ names was assigned on the basis of the academic contribution of each author.

## Supplementary Material

Supplemental data

Supplemental video 1

Supplemental video 10

Supplemental video 11

Supplemental video 12

Supplemental video 13

Supplemental video 14

Supplemental video 2

Supplemental video 3

Supplemental video 4

Supplemental video 5

Supplemental video 6

Supplemental video 7

Supplemental video 8

Supplemental video 9

Supporting data values

## Version 1 12/21/2023

In-Press Preview

## Version 2 03/01/2024

Electronic publication
