# Supplementary material for: Mechanosensitive channels TMEM63A and TMEM63B mediate lung inflation–induced surfactant secretion
Source: J Clin Invest. 2023 Dec 21;134(5):e174508. doi: 10.1172/JCI174508 (PMC10904053; doi:10.1172/JCI174508)
Supplement: Supplemental data [file jci-134-174508-s008.pdf]

Supplemental Figure 1

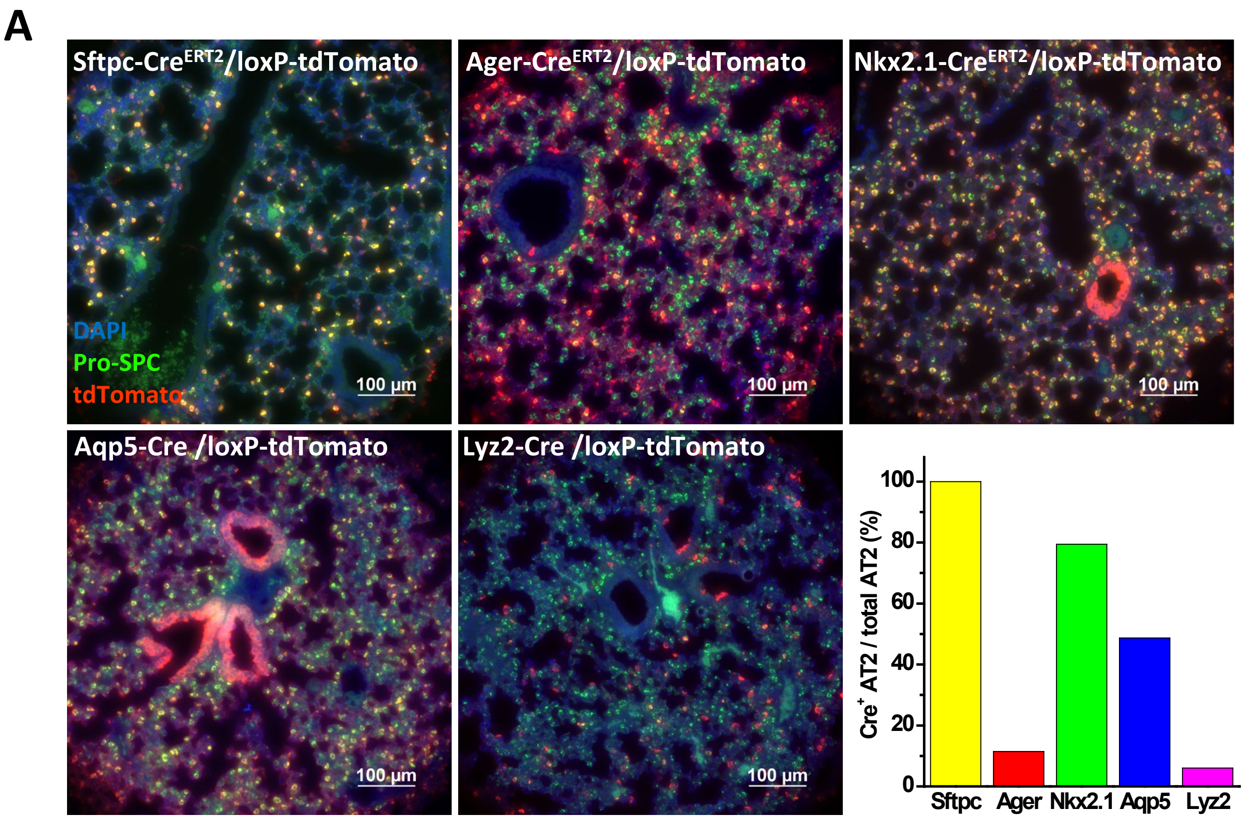

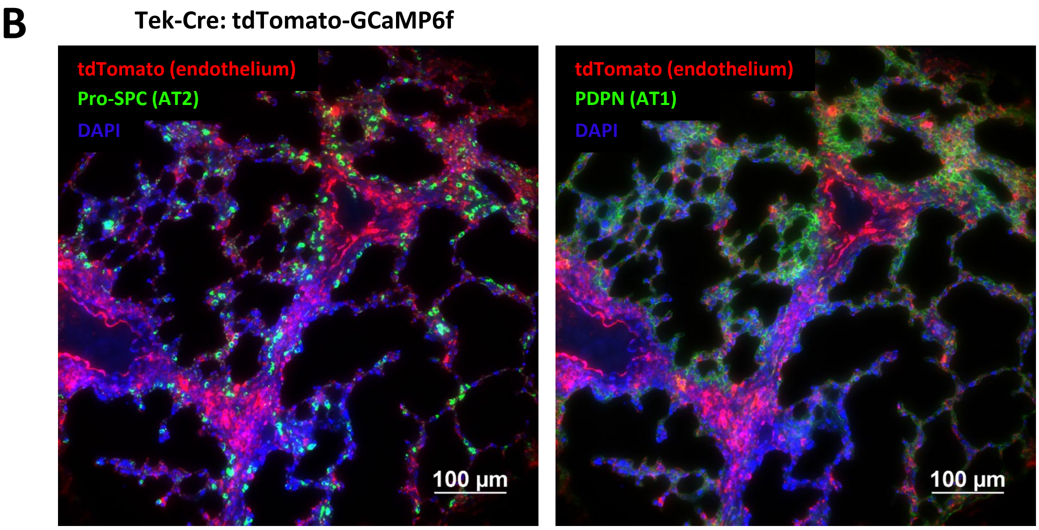

Supplemental Figure 1. Immunofluorescence against Pro-SPC (AT2 marker) and tdTomato in lung sections from adult mice of 2-3 months old.

A, Percentage of Cre^{+} ($tdTomato^{+}$) AT2 cells in total AT2 cells. The expression of tdTomato is dependent on Cre activity in specific cells.

B, Tek-Cre does not induce tdTomato-GCaMP6f expression in AT2 and AT1 cells (no overlapping between tdTomato and Pro-SPC/PDPN).

Supplemental Figure 2

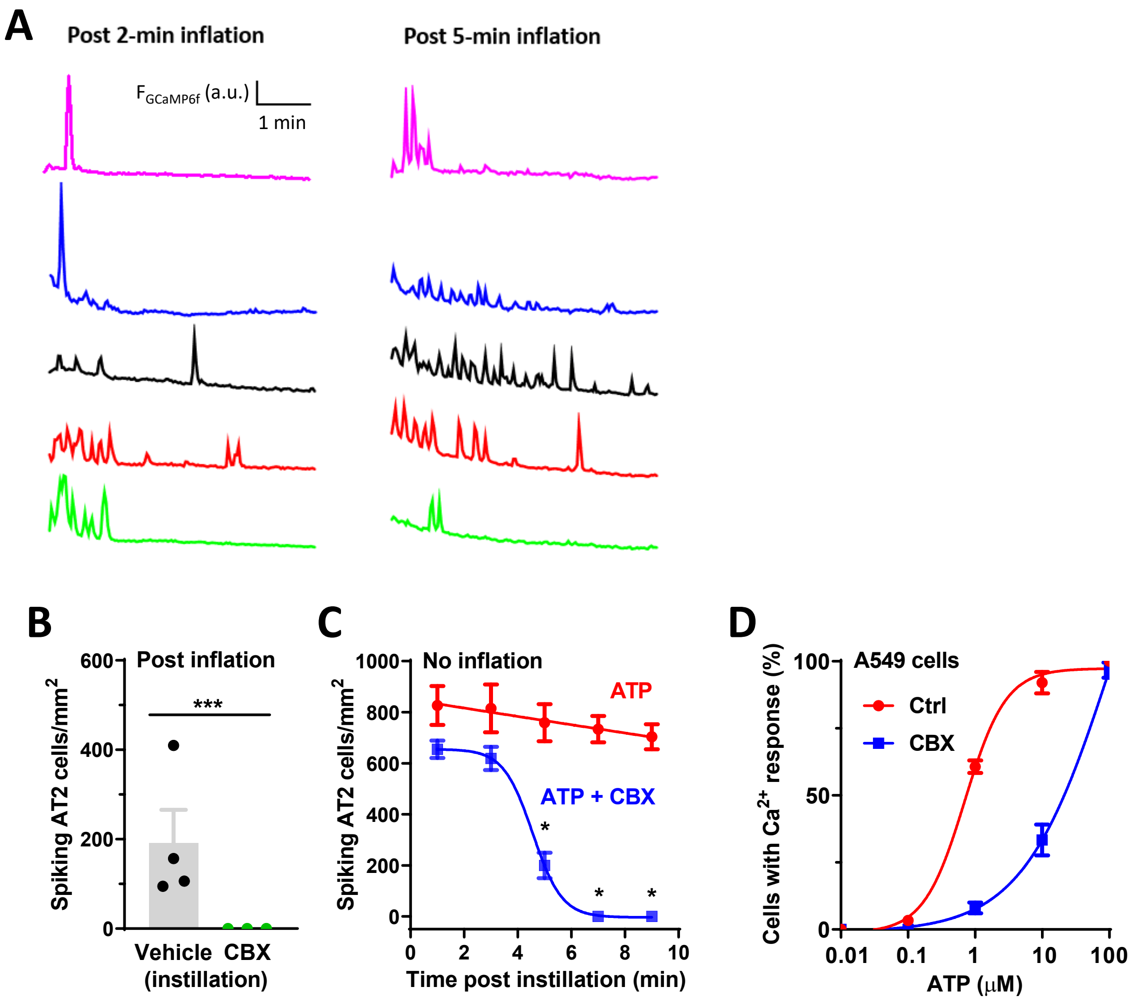

Supplemental Figure 2. Effects of ventilation duration, carbenoxolone and Na^+/K^+ ATPase inhibitors on Ca^{2+} transients in AT2 cells in *ex vivo* mouse lungs.

A, GCaMP6f fluorescence traces of AT2 cells after mechanical ventilation for 2 and 5 min. Each trace represents fluorescence of an AT2 cell.

B, Instillation of 200 μM carbenoxolone (CBX) completely abolished lung inflation-induced Ca^{2+} transients in AT2 cells. *n*=3 lung lobes. $^{***}P<0.001$ by unpaired *t* test.

C, CBX (200 μM) inhibited AT2 Ca^{2+} transients induced by 10 μM ATP instilled in the lungs. The concentration of ATP decreased with time due to hydrolysis on the surface of alveolar cells. The inhibitory action of CBX on purinergic receptors was more potent when the concentration of ATP became lower. *n*=3 lung lobes. $^{*}P<0.05$, two-way ANOVA and Sidak's test.

D, CBX (100 μM) counteracted the effect of ATP at concentrations < 100 μM on purinergic receptors in A549 cells. *n*=3 fields of view; 142, 156 and170 cells/field.

Supplemental Figure 3

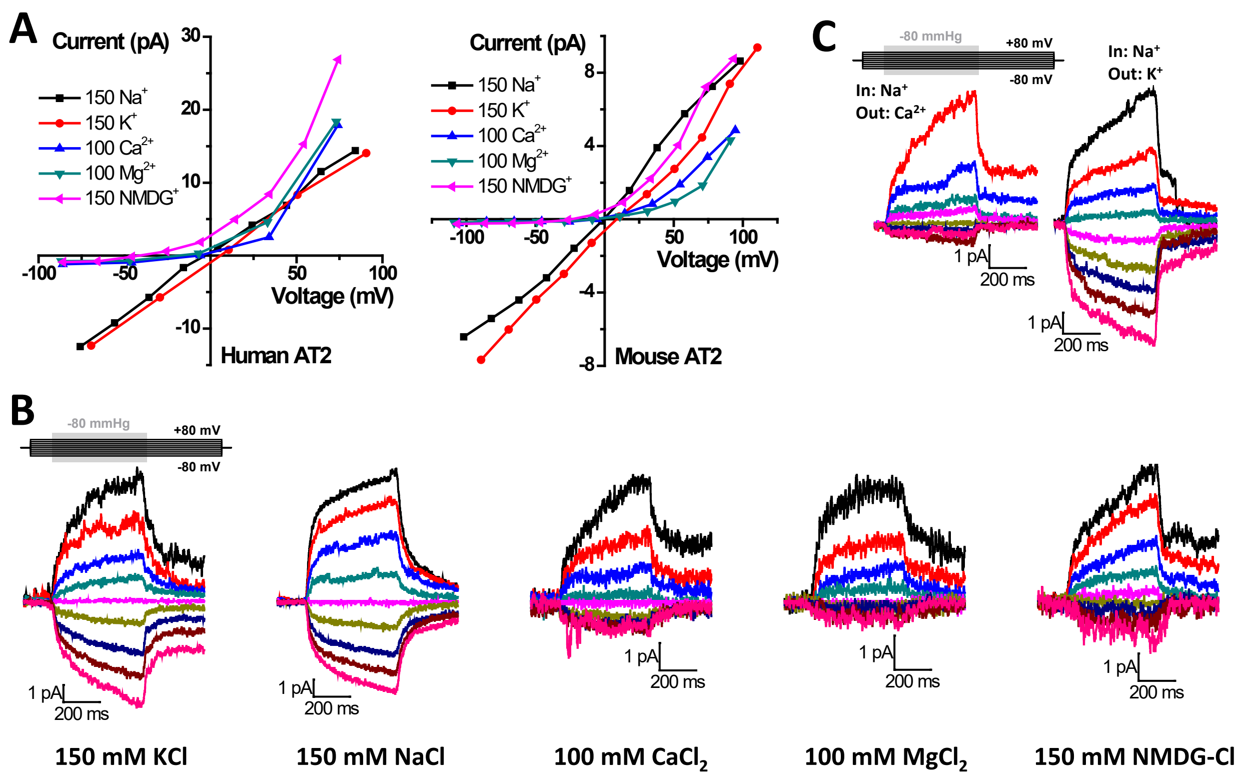

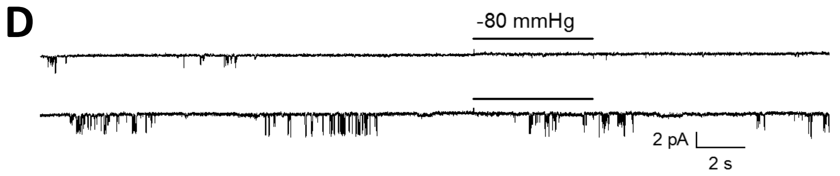

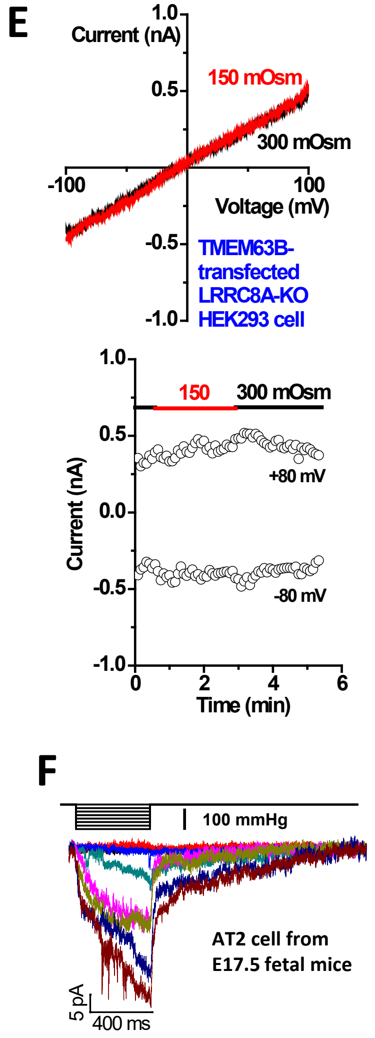

Supplemental Figure 3. High K^{+} and Na^{+} permeability of stretch-activated currents in AT2 cells.

A, Representative current-voltage relationships of -80 mmHg stretch-activated currents recorded with different ions in pipette solution (shown in the figure, mM) under cell-attached configuration from human and mouse AT2 cells.

B, Representative traces of -80 mmHg stretch-activated currents recorded with different ions in pipette solution under cell-attached configuration from mouse AT2 cells.

C, Stretch-activated currents from inside-out recordings showing much higher permeability of Na^{+} and K^{+} than Ca^{2+}. Voltage steps from -80 to +80 mV and vacuum pressure of -80 mmHg were applied to membrane patch from mouse AT2 cells.

D, Epithelial Na^{+} channel (ENaC)-like currents in AT2 cells are insensitive to membrane stretch. Membrane patches with relatively sparse single-channel activities were chosen for vacuum stretch to see potential increase in open probability.

E, No hypotonicity-induced current in TMEM63B-transfented LRRC8A-KO HEK293 cells.

F, Stretch-activated currents in AT2 cells from E17.5 fetal mice.

Supplemental Figure 4

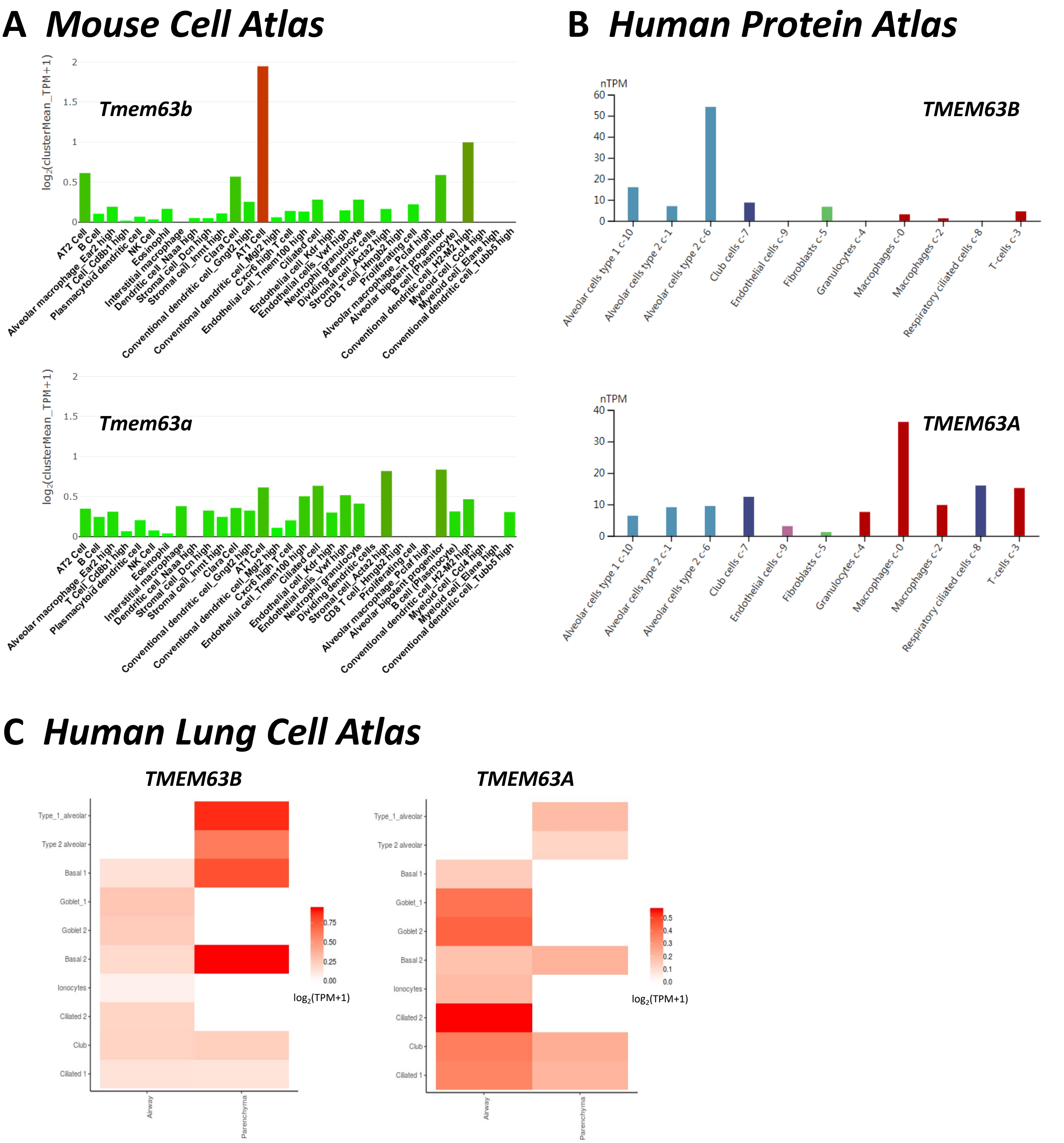

Supplemental Figure 4. Single cell mRNA expression of TMEM63B and TMEM63A in mouse and human lungs. Data retrieved from: **A**, Mouse Cell Atlas (http://bis.zju.edu.cn/MCA/index.html). **B**, Human Protein Atlas (https://www.proteinatlas.org/). **C**, Human Lung Cell Atlas (https://asthma.cellgeni.sanger.ac.uk/).

Supplemental Figure 5

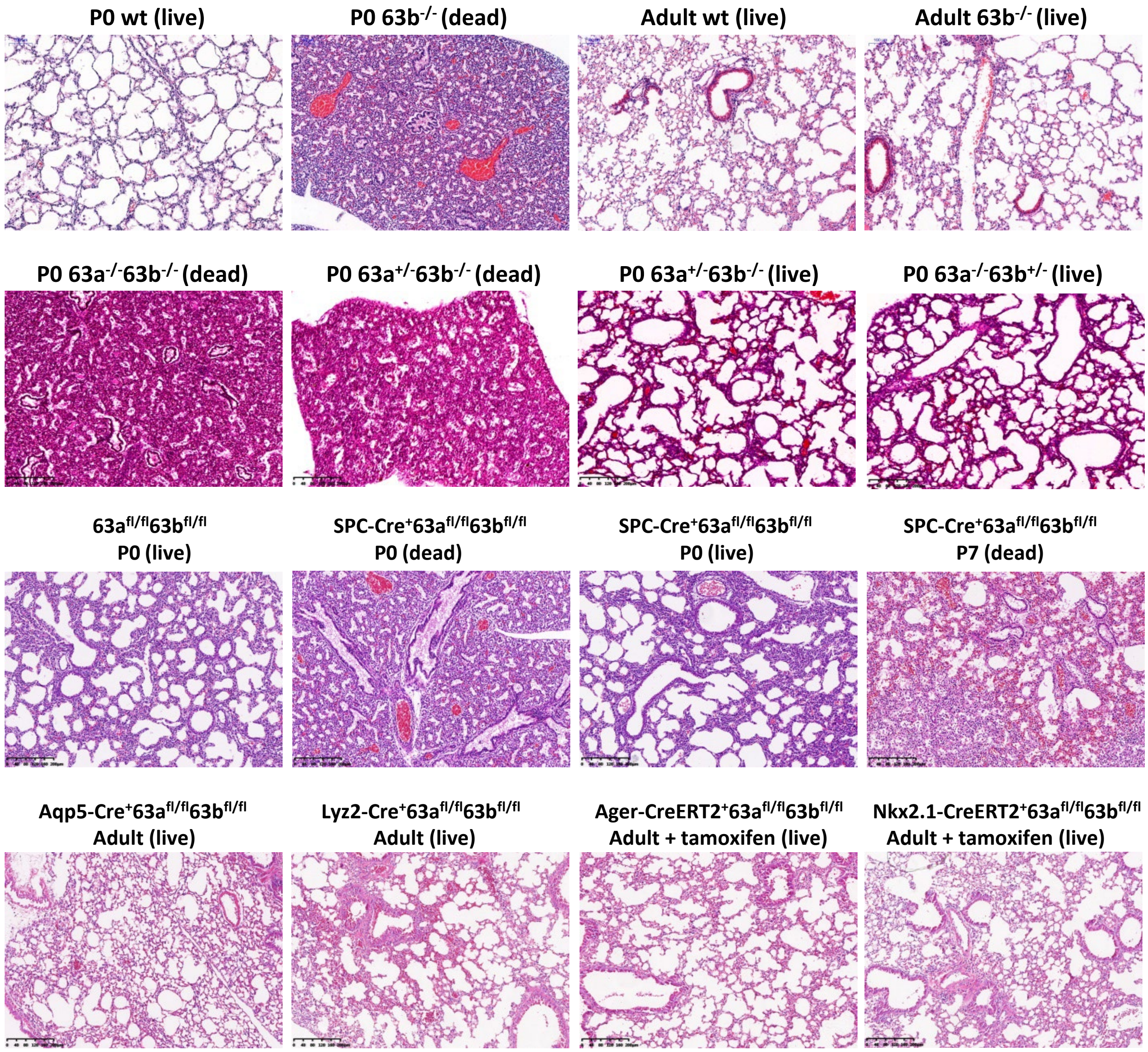

Supplemental Figure 5. HE staining of lung sections from mice with different genotypes and ages.

Supplemental Figure 6

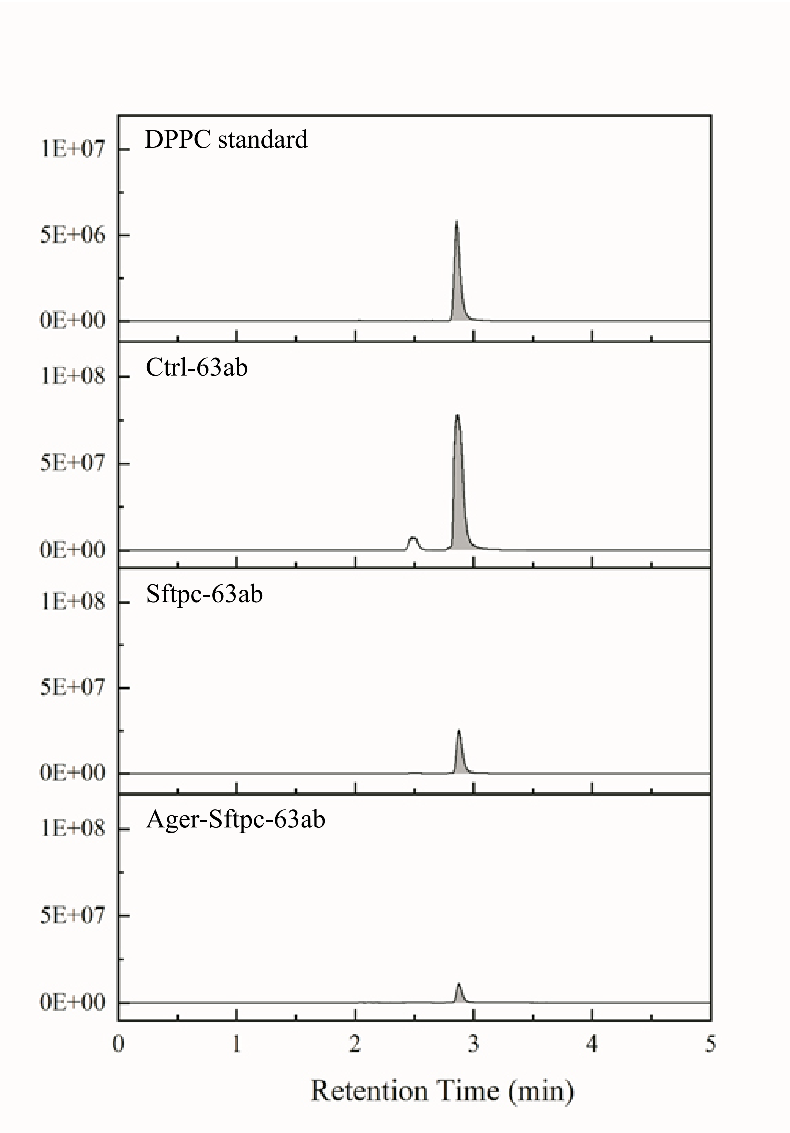

Supplemental Figure 6. The TIC chromatogram of DPPC standard at 2 µg/ml and DPPC in mouse bronchoalverolar lavage fluid by LC-MS/MS.

Supplemental Figure 7

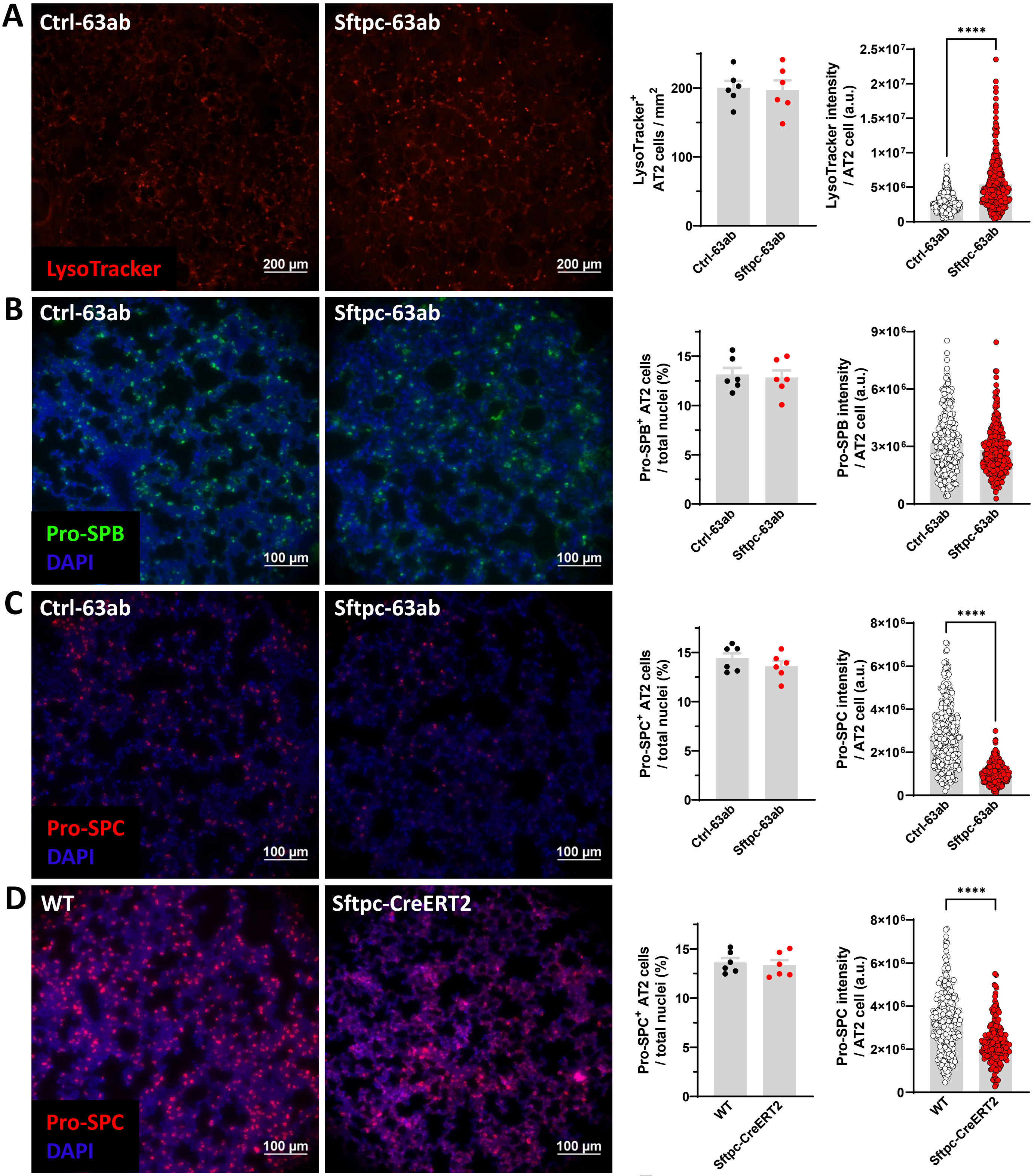

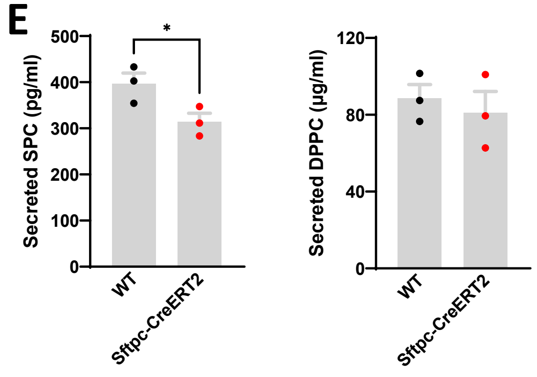

Supplemental Figure 7. Staining of AT2 cells in *Tmem63a/b* cDKO mouse lungs. A, LysoTracker fluorescence in acute lung slices and quantification for AT2 cell density and LysoTracker intensity. *n*=6 lung lobes from 2 mice. **B-D**, Pro-SPB and Pro-SPC immunofluorescence in paraffin-embedded lung sections and quantification for AT2 cell density and Pro-SPB/Pro-SPC intensity. *n*=6 lung lobes from 2 mice. $^{****}P<0.0001$ by unpaired *t* test. **E**, Concentrations of SPC and DPPC in bronchoalveolar lavage fluid of WT and Sftpc-CreERT2 mice. *n*=3 mice. $^{*}P<0.05$ by unpaired *t* test.

Supplemental Figure 8

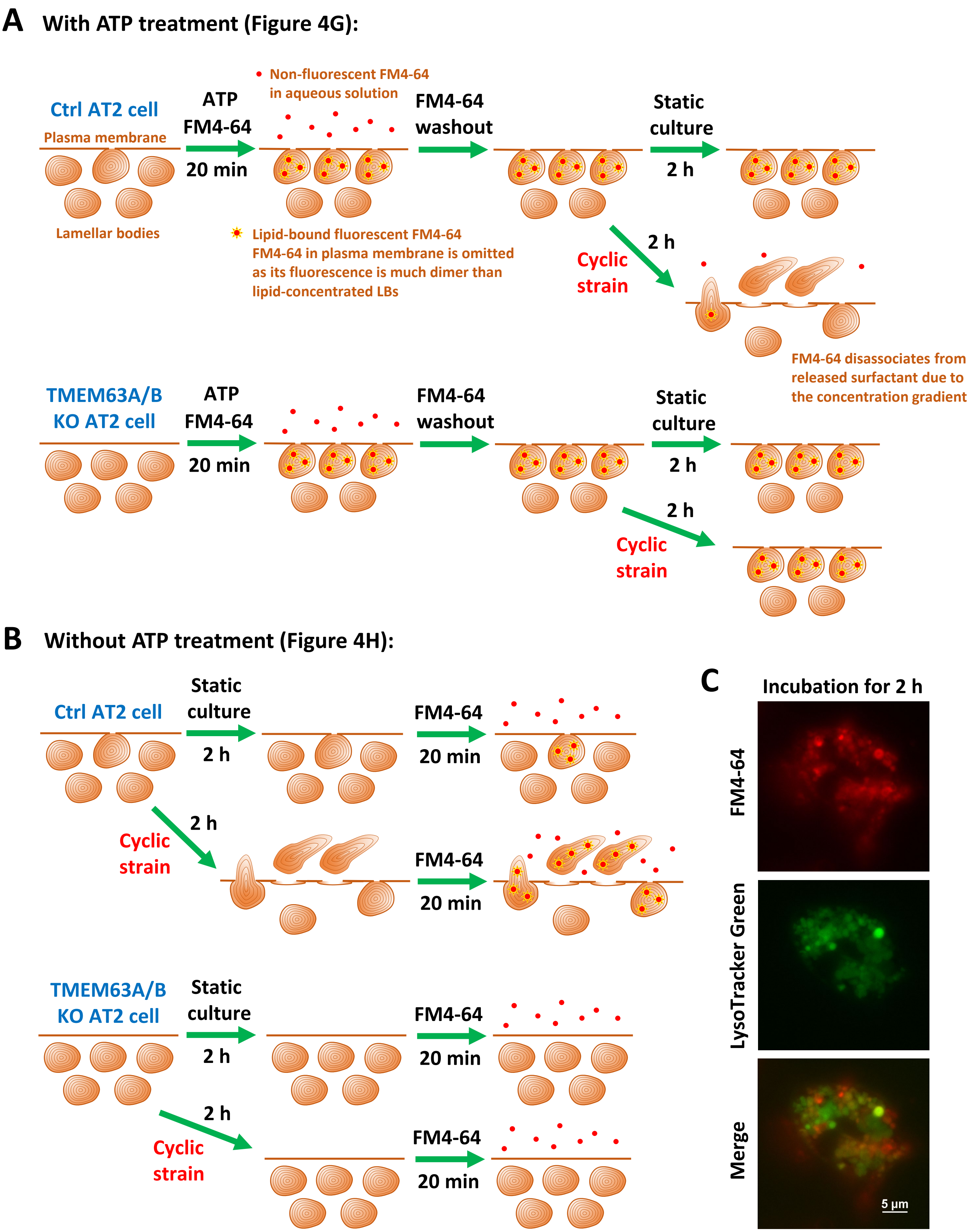

Supplemental Figure 8. Schematic description for cell strain-induced exocytosis of LBs in AT2 cells with (A) or without (B) ATP treatment. Note that FM4-64 cannot be used for long-time incubation as it can be endocytosed and diffuses into all LBs (**C**).

Supplemental Figure 9

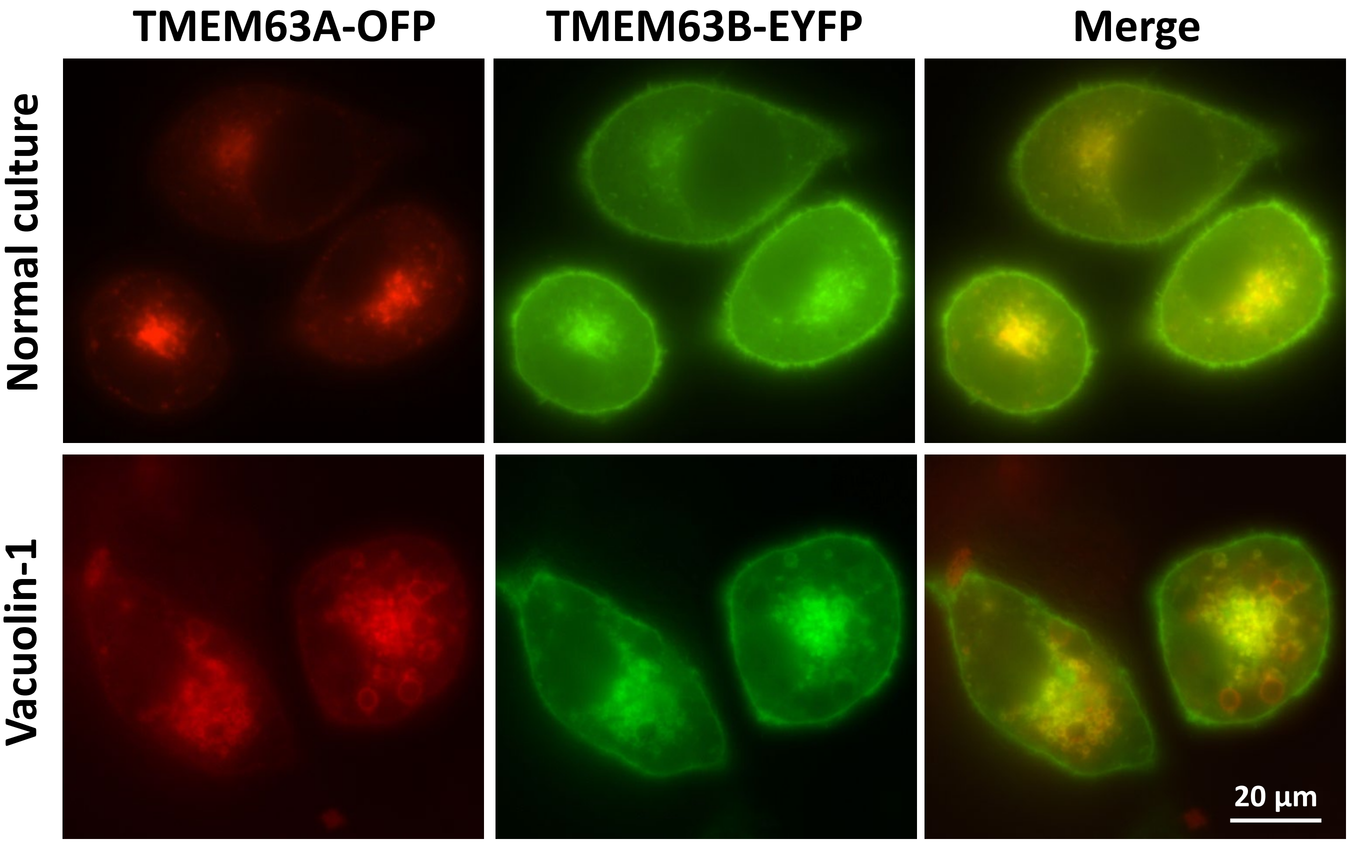

Supplemental Figure 9. Localization of TMEM63A/B in transfected Hela cells in normal culture or treated with vacuolin-1 overnight.

Supplemental Figure 10

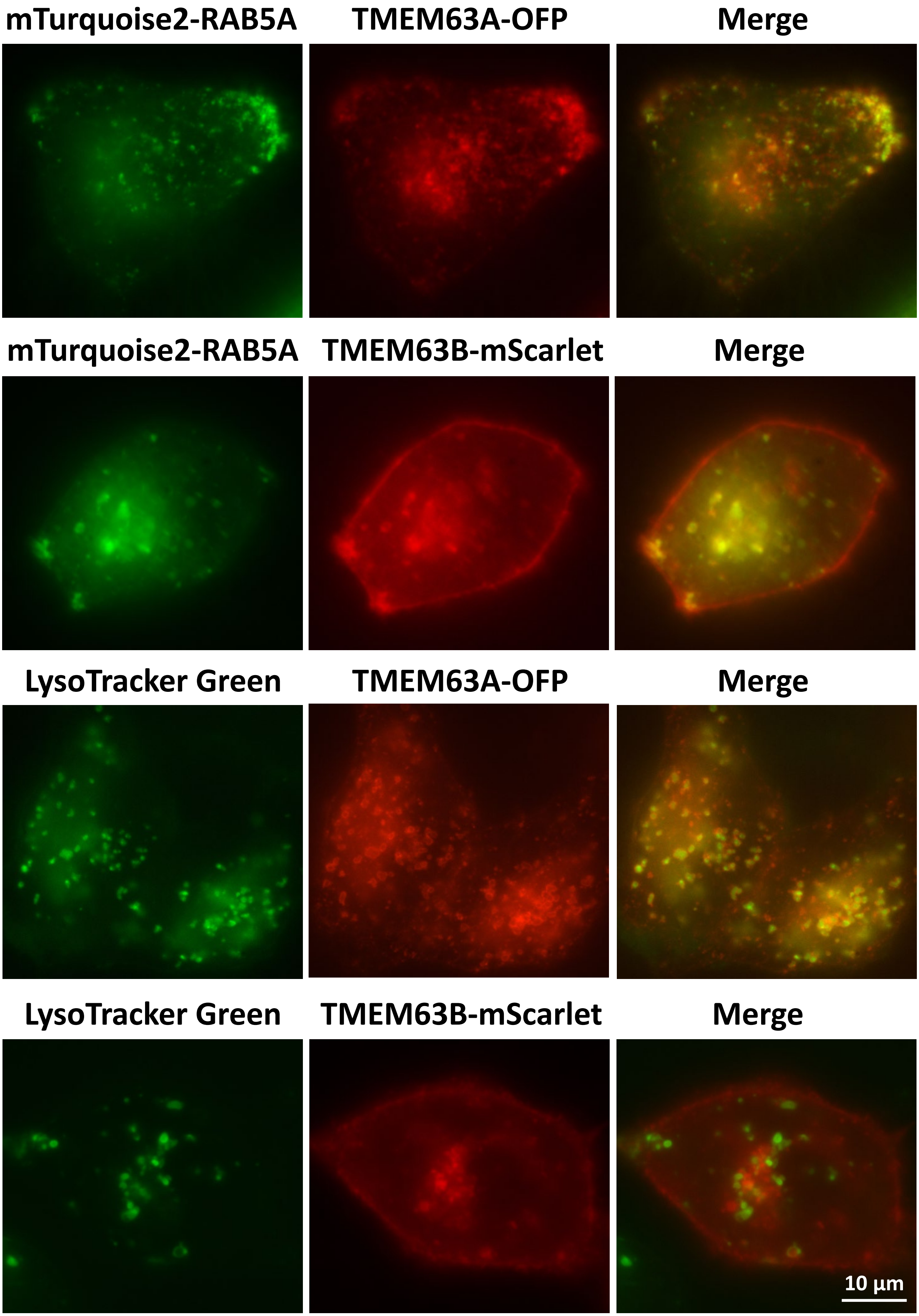

Supplemental Figure 10. Localization of TMEM63A/B in cells co-transfected with RAB5A (marker for early endosome) or stained with Lysotracker Green (lysosome/late endosome).

Supplemental Figure 11

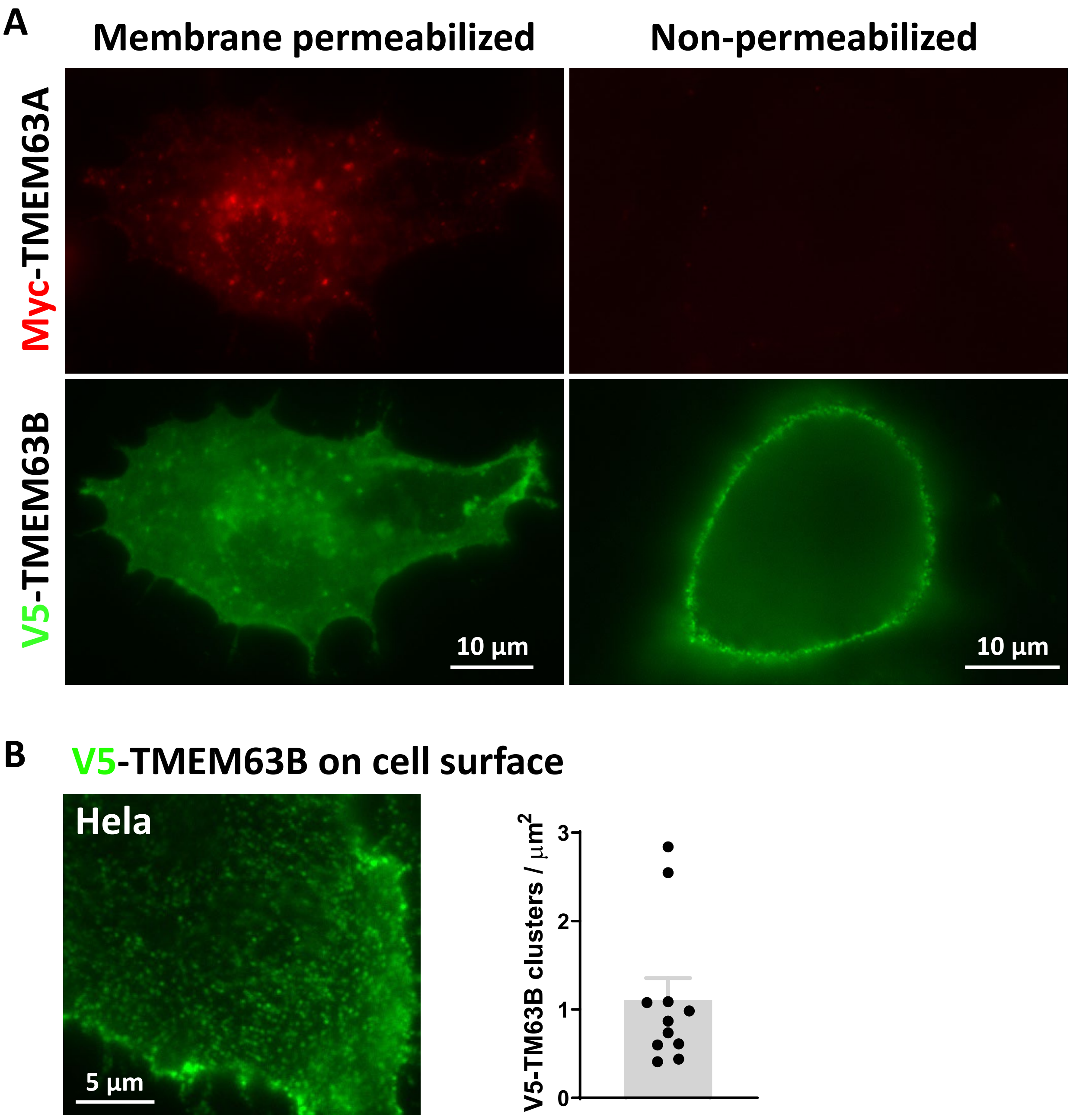

Supplemental Figure 11. TMEM63B is abundantly present at the plasma membrane of transfected Hela cells.
A, Immunofluorescence of Myc-TMEM63A and V5-TMEM63B in membrane permeabilized and non-permeabilized Hela cells. The same batch of Myc-TMEM63A and V5-TMEM63B co-transfected cells were used for staining.
B, TMEM63B clusters on the surface of Hela cells.

Supplemental Figure 12

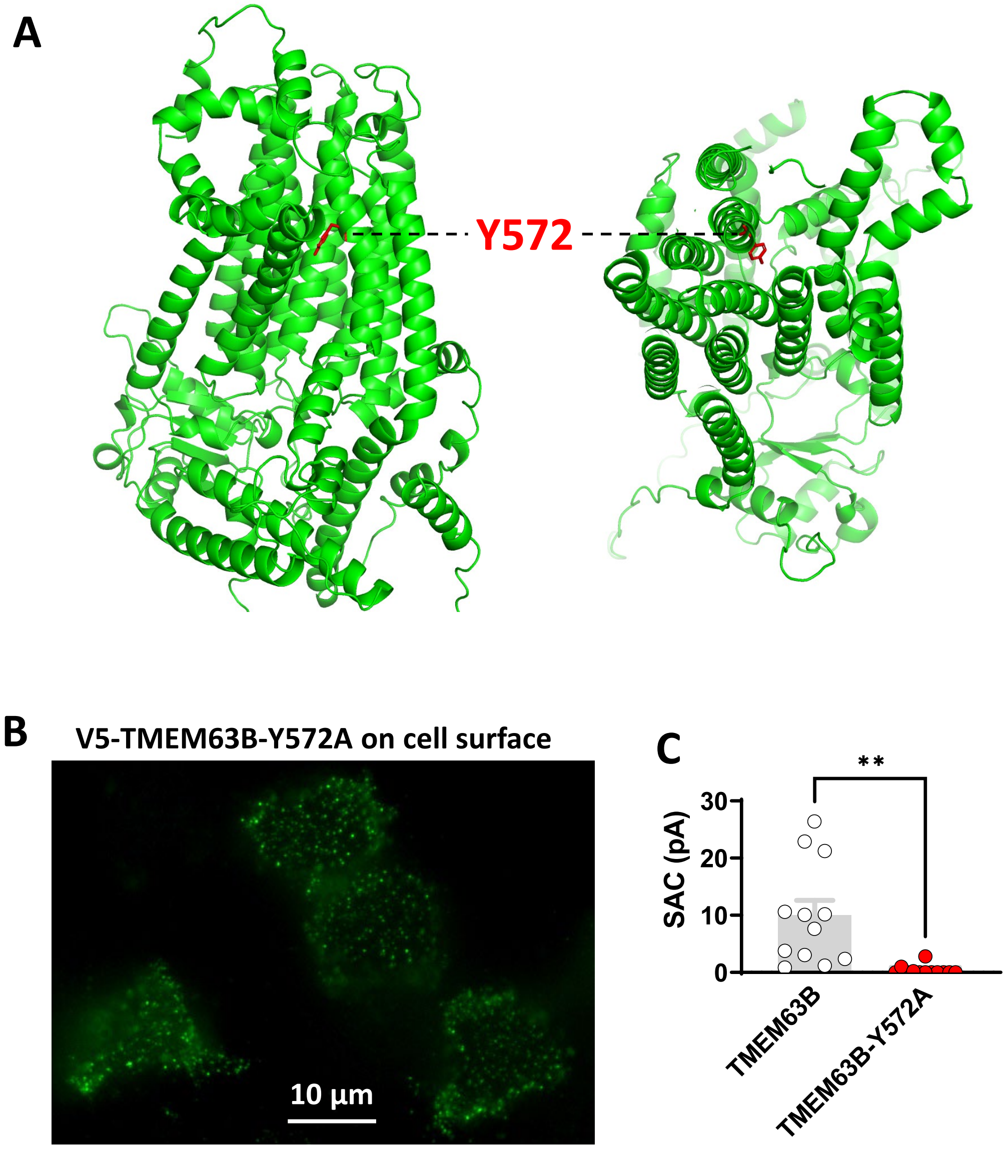

Supplemental Figure 12. Cell surface expression and stretch-insensitivity of TMEM63B-Y572A mutant.

A, Side and top views for the location of Y572 residue in AlphaFold-predicted structure of TMEM63B. The side chain of Y572 is shown in red and facing the putative pore of TMEM63B.

B, Immunofluorescence of V5 tag at the N terminus of TMEM63B-Y572A in non-permeabilized Hela cells.

C, Stretch-activated currents in Hela cells transfected with TMEM63B and TMEM63B-Y572A. Current amplitudes measured at -80 mV and -80 mmHg are shown. n=12 cells; $^{**}P$<0.01 by unpaired t test.

Supplemental Figure 13

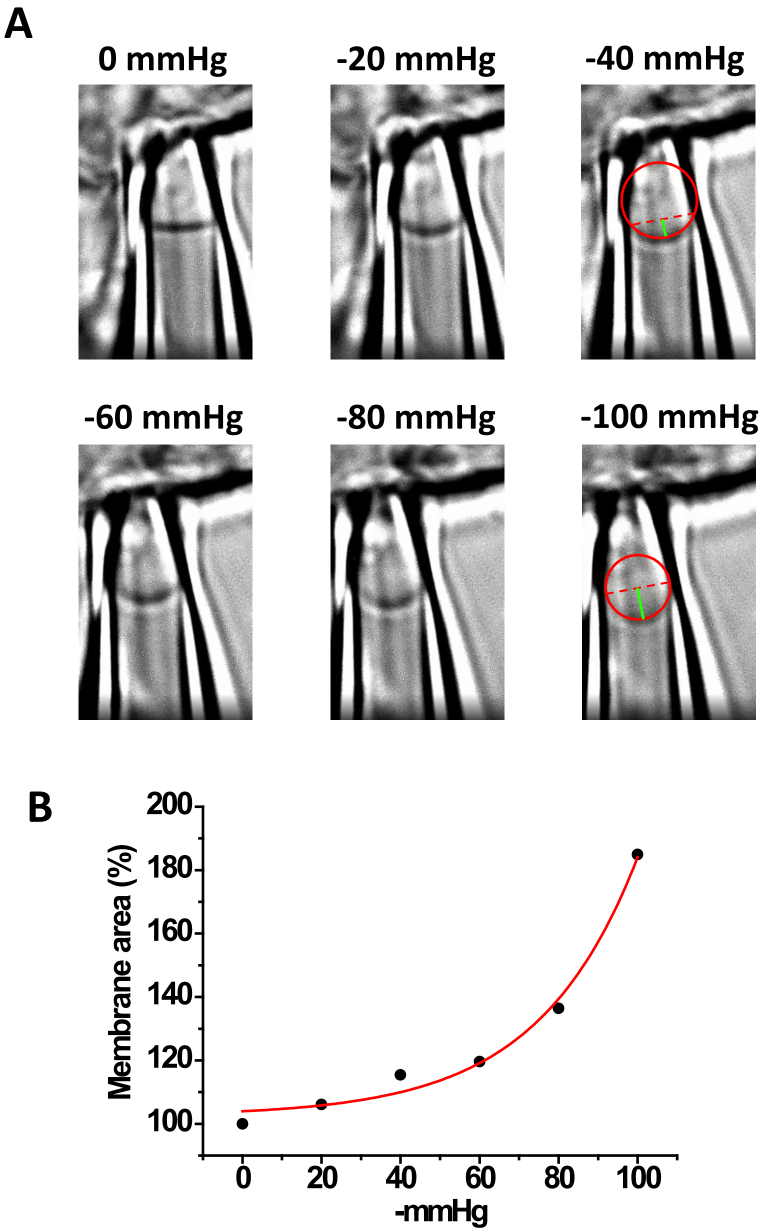

Supplemental Figure 13. Negative pressure-induced membrane area changes in pressure clamp.

A, Shapes of membrane in a micropipette under cell-attached configuration (original video from Lewis & Grandl, Elife 2015; 4: e12088). The membrane curvature is fitted by a circle with variable radius (R). The stretched membrane is considered a spherical cap with a constant diameter (d, dashed line) and different heights (h, green line) in response to different pressures.

B, The areas of membrane calculated with the equation $S=2\pi Rh$ for surface area of a spherical cap. The values were normalized with the membrane area at 0 mmHg as 100%. Data were fitted with a single exponential function.

Supplemental Table 1 Lethality of *Tmem63a/b* knockout in C57BL/6 mice

Genotype	Abbreviation	Tissue/cells affected	KO stage	Lethality		
				P0	P7	P28
Tmem63a$^{-/-}$	63a$^{-/-}$	Whole body	Constitutive	All viable	All viable	All viable
Tmem63b$^{-/-}$	63b$^{-/-}$	Whole body	Constitutive	18% viable	18% viable	18% viable
Tmem63a$^{-/-}$ Tmem63b$^{-/-}$	63a$^{-/-}$63b$^{-/-}$	Whole body	Constitutive	None viable		
Tmem63a$^{+/-}$ Tmem63b$^{-/-}$	63a$^{+/-}$63b$^{-/-}$	Whole body	Constitutive	15% viable	5% viable	5% viable
Tmem63a$^{-/-}$ Tmem63b$^{+/-}$	63a$^{-/-}$63b$^{+/-}$	Whole body	Constitutive	94% viable	94% viable	94% viable
Tmem63a$^{+/-}$ Tmem63b$^{+/-}$	63a$^{+/-}$63b$^{+/-}$	Whole body	Constitutive	All viable	All viable	All viable
Aqp5-Cre$^{+/-}$ Tmem63a$^{fl/fl}$ Tmem63b$^{fl/fl}$	Aqp5-63ab	AT1, ~50% AT2 and club cells	Embryonic	All viable	All viable	All viable
SPC-Cre$^{+/-}$ Tmem63a$^{fl/fl}$ Tmem63b$^{fl/fl}$	SPC-63ab	All AT2 cells	Embryonic	20% viable	None viable	
Lyz2-Cre$^{+/-}$ Tmem63a$^{fl/fl}$ Tmem63b$^{fl/fl}$	Lyz2-63ab	Macrophages and ~6% AT2 cells	Embryonic	All viable	All viable	All viable
Ager-CreERT2$^{+/-}$ Tmem63a$^{fl/fl}$ Tmem63b$^{fl/fl}$	Ager-63ab	~75% AT1 and ~10% AT2 cells	Tamoxifen induction in adults	All viable		
Sftpc-CreERT2$^{+/-}$ Tmem63a$^{fl/fl}$ Tmem63b$^{fl/fl}$	Sftpc-63ab	All AT2 cells	Tamoxifen induction in adults	All died within 10-14 days post tamoxifen administration due to respiratory failure		
Ager-CreERT2$^{+/-}$ Sftpc-CreERT2$^{+/-}$ Tmem63a$^{fl/fl}$ Tmem63b$^{fl/fl}$	Ager-Sftpc-63ab	~75% AT1 and all AT2 cells	Tamoxifen induction in adults	All died within 10-12 days post tamoxifen administration due to respiratory failure		
Nkx2.1-CreERT2$^{+/-}$ Tmem63a$^{fl/fl}$ Tmem63b$^{fl/fl}$	Nkx2.1-63ab	~64% AT1, ~80% AT2 and club cells	Tamoxifen induction in adults	All viable		

Supplemental Table 2 Materials and resources

REGENT or RESOURCE	SOURCE	IDENTIFIER
**Antibodies**		
Rabbit anti-mouse Pro-SPC	Abcam	ab211326
Rat anti-mouse PDPN	Abcam	ab256559
Goat anti-Flag tag	Novus	NB600-344
Goat anti-tdTomato	LSBio	LS-C340696
Rat anti-LAMP1	Santa Cruz	sc-19992
Rabbit anti-V5 tag	CST	13202
Rat anti-Myc tag	Abcam	ab206486
Rabbit anti-Flag tag	CST	14793S
Donkey anti-Rabbit IgG (H+L) Highly Cross-Adsorbed Secondary Antibody, Alexa Fluor™ 488	Thermo Fisher Scientific	A-21206
Donkey anti-Goat IgG (H+L) Highly Cross-Adsorbed Secondary Antibody, Alexa Fluor™ Plus 555	Thermo Fisher Scientific	A32816
Donkey anti-Rat IgG (H+L) Highly Cross-Adsorbed Secondary Antibody, Alexa Fluor™ Plus 647	Thermo Fisher Scientific	A48272
**Chemicals, peptides, and recombinant proteins**		
NaCl	Sangon Biotech	A100241; CAS: 7647-14-5
KCl	Sangon Biotech	A100395; CAS: 7447-40-7
$CaCl_2$	Sigma-Aldrich	21115; CAS: 10043-52-4
$MgCl_2$	Sangon Biotech	A601336; CAS: 7791-18-6
CsCl	aladdin	C105368; CAS: 7647-17-8
HEPES	Sangon Biotech	A100511; CAS: 7365-45-9
D-glucose	Sigma-Aldrich	G7021; CAS: 50-99-7
EGTA	Sangon Biotech	A600077; CAS:67-42-5
N-methyl-D-glucamine (NMDG)	D&B Biological	H824001; CAS: 6284-40-8
Na_2ATP	Sigma-Aldrich	A26209; CAS: 34369-07-8
NaOH	Sangon Biotech	A100583; CAS: 1310-73-2
KOH	Sangon Biotech	A610441; CAS: 1310-58-3
CsOH	Sigma-Aldrich	232041; CAS:21351-79-1
Fluo-4 AM	Thermo Fisher Scientific	F14201
FM4-64	Biotium	70021
LysoTracker Green	Beyotime	C1047S
LysoTracker Red	BBI Life Sciences	E607506
Dimethyl sulfoxide (DMSO)	Thermo Scientific	20688
Suramin	aladdin	S131869; CAS: 129-46-4

Apyrase	Sigma-Aldrich	A7646; CAS: 9000-95-7
U-73122	MedChemExpress	HY-13419; CAS: 112648-68-7
Carbenoxolone	aladdin	C185282; CAS: 5697-56-3
Gap26	MedChemExpress	HY-P1082; CAS: 197250-15-0
Probenecid	Sigma-Aldrich	P8761; CAS: 57-66-9
DIDS	MedChemExpress	HY-D0086; CAS: 67483-13-0
Clodronate	Macklin	D834709; CAS: 88416-50-6
Amiloride	MedChemExpress	HY-B0285; CAS: 2609-46-3
Quinine	BBI Life Sciences	A602122; CAS:130-95-0
Ruthenium red	Macklin	R817195; CAS: 11103-72-3
Vacuolin-1	MedChemExpress	HY-118630; CAS: 351986-85-1
Tamoxifen	D&B Biological	K901668; CAS: 10540-29-1
Isoflurane	RWD	R510-2; CAS: 832740-98-4
Ouabain	MedChemExpress	HY-B0542; CAS: 11018-89-6
Digoxin	AbMole	M3935; CAS: 20830-75-5
Fibronectin	SAITONG	H10049
Lipofectamine 2000	Thermo Fisher Scientific	11668019
DMEM/F-12	Gibco	10565-018
Phosphate-buffered saline (PBS)	ORIGENE	ZLI-9061
Fetal bovine serum (FBS)	Gibco	10270-106
Penicillin and streptomycin	Beyotime	C0222
Dispase	Coolaber	CD4691
Low melting point agarose	BBI Life Sciences	A600015; CAS: 9012-36-6
DNase I	G-CLONE	EZ0380-Y
Trypsin	Beyotime	C0201
$MgSO_4$	aladdin	M110770; CAS: 10034-99-8
NaH_2PO_4	aladdin	S102313; CAS: 13472-35-0
$NaHCO_3$	aladdin	S112331; CAS: 144-55-8
**Critical commercial assays**		
Mouse SFTPC ELISA Kit	Abcam	ab252366
ATP Determination Kit	Beyotime	S0026
**Biological Samples**		
Human AT2 cells from lung cancer patients	Affiliated Traditional Chinese Medicine Hospital of Southwest Medical University	N/A
AAV: U6-spgRNA(Tmem63b)-donor(V5tag)	OBiO Technology	Custom
AAV: CMV-hTMEM63A-3xFlag-tWPA	OBiO Technology	Custom
AAV: CMV-hTMEM63B-3xFlag-tWPA	OBiO Technology	Custom

AAV: CMV-hTMEM63B-Y572A-3xFlag-tWPA	OBiO Technology	Custom
AAV: CMV-MCS-3xFlag-tWPA	OBiO Technology	Custom
AAV: CAG-DIO-jGCaMP7s-mCherry-WPRE	OBiO Technology	Custom
AAV: CMV-DIO-EGFP-WPRE	OBiO Technology	Custom
**Experimental models: Cell lines**		
Human: Hela	ATCC	CCL-2
Human: A549	ATCC	CCL-185
Human: LRRC8A-KO HEK293	David Clapham Lab	N/A
**Experimental models: Organisms/Strains**		
Mouse: C57BL/6N-Tmem63a-KO	Cyagen	S-KO-04604
Mouse: C57BL/6N-Tmem63b-KO	Cyagen	S-KO-05840
Mouse: C57BL/6N-Tmem63a-cKO	Cyagen	S-CKO-05381
Mouse: C57BL/6N-Tmem63b-cKO	Cyagen	S-CKO-06783
Mouse: C57BL/6-Aqp5$^{em1(iCre-WPRE-polyA)Smoc}$	Shanghai Model Organisms Center	NM-KI-200062
Mouse: B6/JGpt-Sftpc$^{em1Cin(IRES-iCre)}$/Gpt	GemPharmatech	T004715
Mouse: B6.129P2-Lyz2$^{tm1(cre)Ifo}$/J	The Jackson Laboratory	004781
Mouse: B6.Cg-Ager$^{tm2.1(cre/ERT2)Blh}$/2J	The Jackson Laboratory	032771
Mouse: B6.129S-Sftpc$^{tm1(cre/ERT2)Blh}$/J	The Jackson Laboratory	028054
Mouse: Nkx2-1$^{tm1.1(cre/ERT2)Zjh}$/J	The Jackson Laboratory	014552
Mouse: B6.Cg-Tg(Tek-cre)12Flv/J	The Jackson Laboratory	004128
Mouse: B6.129P2(Cg)-Cx3cr1$^{tm2.1(cre/ERT2)Litt}$/WganJ	The Jackson Laboratory	021160
Mouse: B6(129S4)-Gt(ROSA)26Sor$^{tm1.1(CAG-tdTomato/GCaMP6f)Mdcah}$/J	The Jackson Laboratory	031968
Mouse: C57BL/6J-ROSA26-Cas9	Cyagen	C001218
**Recombinant DNA**		
pCMV-TMEM63A-OFP	SinoBiological	MG51287-ACR
pcDNA4/TO-Myc-TMEM63A	This study	N/A
pcDNA4/TO-V5-TMEM63B	This study	N/A
pcDNA4/TO-V5-TMEM63B-Y572A	This study	N/A
pcDNA4/TO-TMEM63B-EYFP	This study	N/A
pcDNA3.1-mTurquoise2-RAB5A	This study	N/A
pcDNA3.1-TMEM63B-mScarlet	This study	N/A
**Software and Algorithms**		

NIS-Elements AR 4.30	Nikon	https://www.nikon.com/
pClamp 10.6	Molecular Devices	https://www.moleculardevices.com/
PatchMaster 2x90.5	HEKA	https://www.elproscan.com/
GraphPad Prism 9	GraphPad	https://www.graphpad.com/
OriginPro 8	OriginLab	https://www.originlab.com/

Supplemental Table 1 Lethality of *Tmem63a/b* knockout in C57BL/6 mice

Genotype	Abbreviation	Tissue/cells affected	KO stage	Lethality		
				**P0**	**P7**	**P28**
Tmem63a$^{-/-}$	63a$^{-/-}$	Whole body	Constitutive	All viable	All viable	All viable
Tmem63b$^{-/-}$	63b$^{-/-}$	Whole body	Constitutive	18% viable	18% viable	18% viable
Tmem63a$^{-/-}$ Tmem63b$^{-/-}$	63a$^{-/-}$63b$^{-/-}$	Whole body	Constitutive	None viable		
Tmem63a$^{+/-}$ Tmem63b$^{-/-}$	63a$^{+/-}$63b$^{-/-}$	Whole body	Constitutive	15% viable	5% viable	5% viable
Tmem63a$^{-/-}$ Tmem63b$^{+/-}$	63a$^{-/-}$63b$^{+/-}$	Whole body	Constitutive	94% viable	94% viable	94% viable
Tmem63a$^{+/-}$ Tmem63b$^{+/-}$	63a$^{+/-}$63b$^{+/-}$	Whole body	Constitutive	All viable	All viable	All viable
Aqp5-Cre$^{+/-}$ Tmem63a$^{fl/fl}$ Tmem63b$^{fl/fl}$	Aqp5-63ab	AT1, ~50% AT2 and club cells	Embryonic	All viable	All viable	All viable
SPC-Cre$^{+/-}$ Tmem63a$^{fl/fl}$ Tmem63b$^{fl/fl}$	SPC-63ab	All AT2 cells	Embryonic	20% viable	None viable	
Lyz2-Cre$^{+/-}$ Tmem63a$^{fl/fl}$ Tmem63b$^{fl/fl}$	Lyz2-63ab	Macrophages and ~6% AT2 cells	Embryonic	All viable	All viable	All viable
Ager-CreERT2$^{+/-}$ Tmem63a$^{fl/fl}$ Tmem63b$^{fl/fl}$	Ager-63ab	~75% AT1 and ~10% AT2 cells	Tamoxifen induction in adults	All viable		
Sftpc-CreERT2$^{+/-}$ Tmem63a$^{fl/fl}$ Tmem63b$^{fl/fl}$	Sftpc-63ab	All AT2 cells	Tamoxifen induction in adults	All died within 10-14 days post tamoxifen administration due to respiratory failure		
Ager-CreERT2$^{+/-}$ Sftpc-CreERT2$^{+/-}$ Tmem63a$^{fl/fl}$ Tmem63b$^{fl/fl}$	Ager-Sftpc-63ab	~75% AT1 and all AT2 cells	Tamoxifen induction in adults	All died within 10-12 days post tamoxifen administration due to respiratory failure		
Nkx2.1-CreERT2$^{+/-}$ Tmem63a$^{fl/fl}$ Tmem63b$^{fl/fl}$	Nkx2.1-63ab	~64% AT1, ~80% AT2 and club cells	Tamoxifen induction in adults	All viable		

Supplemental Table 2 Materials and resources

REGENT or RESOURCE	SOURCE	IDENTIFIER
**Antibodies**		
Rabbit anti-mouse Pro-SPC	Abcam	ab211326
Rat anti-mouse PDPN	Abcam	ab256559
Goat anti-Flag tag	Novus	NB600-344
Goat anti-tdTomato	LSBio	LS-C340696
Rat anti-LAMP1	Santa Cruz	sc-19992
Rabbit anti-V5 tag	CST	13202
Rat anti-Myc tag	Abcam	ab206486
Rabbit anti-Flag tag	CST	14793S
Donkey anti-Rabbit IgG (H+L) Highly Cross-Adsorbed Secondary Antibody, Alexa Fluor™ 488	Thermo Fisher Scientific	A-21206
Donkey anti-Goat IgG (H+L) Highly Cross-Adsorbed Secondary Antibody, Alexa Fluor™ Plus 555	Thermo Fisher Scientific	A32816
Donkey anti-Rat IgG (H+L) Highly Cross-Adsorbed Secondary Antibody, Alexa Fluor™ Plus 647	Thermo Fisher Scientific	A48272
**Chemicals, peptides, and recombinant proteins**		
NaCl	Sangon Biotech	A100241; CAS: 7647-14-5
KCl	Sangon Biotech	A100395; CAS: 7447-40-7
$CaCl_2$	Sigma-Aldrich	21115; CAS: 10043-52-4
$MgCl_2$	Sangon Biotech	A601336; CAS: 7791-18-6
CsCl	aladdin	C105368; CAS: 7647-17-8
HEPES	Sangon Biotech	A100511; CAS: 7365-45-9
D-glucose	Sigma-Aldrich	G7021; CAS: 50-99-7
EGTA	Sangon Biotech	A600077; CAS:67-42-5
N-methyl-D-glucamine (NMDG)	D&B Biological	H824001; CAS: 6284-40-8
Na_2ATP	Sigma-Aldrich	A26209; CAS: 34369-07-8
NaOH	Sangon Biotech	A100583; CAS: 1310-73-2
KOH	Sangon Biotech	A610441; CAS: 1310-58-3
CsOH	Sigma-Aldrich	232041; CAS:21351-79-1
Fluo-4 AM	Thermo Fisher Scientific	F14201
FM4-64	Biotium	70021
LysoTracker Green	Beyotime	C1047S
LysoTracker Red	BBI Life Sciences	E607506
Dimethyl sulfoxide (DMSO)	Thermo Scientific	20688
Suramin	aladdin	S131869; CAS: 129-46-4

Apyrase	Sigma-Aldrich	A7646; CAS: 9000-95-7
U-73122	MedChemExpress	HY-13419; CAS: 112648-68-7
Carbenoxolone	aladdin	C185282; CAS: 5697-56-3
Gap26	MedChemExpress	HY-P1082; CAS: 197250-15-0
Probenecid	Sigma-Aldrich	P8761; CAS: 57-66-9
DIDS	MedChemExpress	HY-D0086; CAS: 67483-13-0
Clodronate	Macklin	D834709; CAS: 88416-50-6
Amiloride	MedChemExpress	HY-B0285; CAS: 2609-46-3
Quinine	BBI Life Sciences	A602122; CAS:130-95-0
Ruthenium red	Macklin	R817195; CAS: 11103-72-3
Vacuolin-1	MedChemExpress	HY-118630; CAS: 351986-85-1
Tamoxifen	D&B Biological	K901668; CAS: 10540-29-1
Isoflurane	RWD	R510-2; CAS: 832740-98-4
Ouabain	MedChemExpress	HY-B0542; CAS: 11018-89-6
Digoxin	AbMole	M3935; CAS: 20830-75-5
Fibronectin	SAITONG	H10049
Lipofectamine 2000	Thermo Fisher Scientific	11668019
DMEM/F-12	Gibco	10565-018
Phosphate-buffered saline (PBS)	ORIGENE	ZLI-9061
Fetal bovine serum (FBS)	Gibco	10270-106
Penicillin and streptomycin	Beyotime	C0222
Dispase	Coolaber	CD4691
Low melting point agarose	BBI Life Sciences	A600015; CAS: 9012-36-6
DNase I	G-CLONE	EZ0380-Y
Trypsin	Beyotime	C0201
$MgSO_4$	aladdin	M110770; CAS: 10034-99-8
NaH_2PO_4	aladdin	S102313; CAS: 13472-35-0
$NaHCO_3$	aladdin	S112331; CAS: 144-55-8
**Critical commercial assays**		
Mouse SFTPC ELISA Kit	Abcam	ab252366
ATP Determination Kit	Beyotime	S0026
**Biological Samples**		
Human AT2 cells from lung cancer patients	Affiliated Traditional Chinese Medicine Hospital of Southwest Medical University	N/A
AAV: U6-spgRNA(Tmem63b)-donor(V5tag)	OBiO Technology	Custom
AAV: CMV-hTMEM63A-3xFlag-tWPA	OBiO Technology	Custom
AAV: CMV-hTMEM63B-3xFlag-tWPA	OBiO Technology	Custom

AAV: CMV-hTMEM63B-Y572A-3xFlag-tWPA	OBiO Technology	Custom
AAV: CMV-MCS-3xFlag-tWPA	OBiO Technology	Custom
AAV: CAG-DIO-jGCaMP7s-mCherry-WPRE	OBiO Technology	Custom
AAV: CMV-DIO-EGFP-WPRE	OBiO Technology	Custom
**Experimental models: Cell lines**		
Human: Hela	ATCC	CCL-2
Human: A549	ATCC	CCL-185
Human: LRRC8A-KO HEK293	David Clapham Lab	N/A
**Experimental models: Organisms/Strains**		
Mouse: C57BL/6N-Tmem63a-KO	Cyagen	S-KO-04604
Mouse: C57BL/6N-Tmem63b-KO	Cyagen	S-KO-05840
Mouse: C57BL/6N-Tmem63a-cKO	Cyagen	S-CKO-05381
Mouse: C57BL/6N-Tmem63b-cKO	Cyagen	S-CKO-06783
Mouse: C57BL/6-Aqp5$^{em1(iCre\text{-}WPRE\text{-}polyA)Smoc}$	Shanghai Model Organisms Center	NM-KI-200062
Mouse: B6/JGpt-Sftpc$^{em1Cin(IRES\text{-}iCre)}$/Gpt	GemPharmatech	T004715
Mouse: B6.129P2-Lyz2$^{tm1(cre)Ifo}$/J	The Jackson Laboratory	004781
Mouse: B6.Cg-Ager$^{tm2.1(cre/ERT2)Blh}$/2J	The Jackson Laboratory	032771
Mouse: B6.129S-Sftpc$^{tm1(cre/ERT2)Blh}$/J	The Jackson Laboratory	028054
Mouse: Nkx2-1$^{tm1.1(cre/ERT2)Zjh}$/J	The Jackson Laboratory	014552
Mouse: B6.Cg-Tg(Tek-cre)12Flv/J	The Jackson Laboratory	004128
Mouse: B6.129P2(Cg)-Cx3cr1$^{tm2.1(cre/ERT2)Litt}$/WganJ	The Jackson Laboratory	021160
Mouse: B6(129S4)-Gt(ROSA)26Sor $^{tm1.1(CAG\text{-}tdTomato/GCaMP6f)Mdcah}$/J	The Jackson Laboratory	031968
Mouse: C57BL/6J-ROSA26-Cas9	Cyagen	C001218
**Recombinant DNA**		
pCMV-TMEM63A-OFP	SinoBiological	MG51287-ACR
pcDNA4/TO-Myc-TMEM63A	This study	N/A
pcDNA4/TO-V5-TMEM63B	This study	N/A
pcDNA4/TO-V5-TMEM63B-Y572A	This study	N/A
pcDNA4/TO-TMEM63B-EYFP	This study	N/A
pcDNA3.1-mTurquoise2-RAB5A	This study	N/A
pcDNA3.1-TMEM63B-mScarlet	This study	N/A
**Software and Algorithms**		

NIS-Elements AR 4.30	Nikon	https://www.nikon.com/
pClamp 10.6	Molecular Devices	https://www.moleculardevices.com/
PatchMaster 2x90.5	HEKA	https://www.elproscan.com/
GraphPad Prism 9	GraphPad	https://www.graphpad.com/
OriginPro 8	OriginLab	https://www.originlab.com/
